# Zipper-interacting protein kinase mediates neuronal cell death and cognitive dysfunction in traumatic brain injury via regulating DEDD

**DOI:** 10.1038/s41419-025-07474-7

**Published:** 2025-03-04

**Authors:** Yingxue Mei, Fei She, Ling Zhang, Gamin Kim, Ruomeng Li, Xiuzhi Zheng, Zonghai Wang, Renxuan Chen, Long Wang, Dongmei Chen, Jungho Kim, Tao Zhang, Tae Ho Lee

**Affiliations:** 1 Fujian Key Laboratory of Translational Research in Cancer and Neurodegenerative Diseases, Institute of Basic Medicine, School of Basic Medical Sciences, Fujian Medical University, Fuzhou, China; 2 Laboratory of Molecular and Cellular Biology, Department of Life Science, Sogang University, Seoul, Korea

**Keywords:** Cell death in the nervous system, Brain injuries

## Abstract

Neuronal cell death is a causative process in traumatic brain injury (TBI)-induced structural and functional impairment of the central nervous system. However, the upstream trigger of TBI-induced neuronal loss and the underlying molecular pathways remain unclear. Zipper-interacting protein kinase (ZIPK) has been shown to be upregulated in Alzheimer’s disease and ischemic stroke and to play a role in cellular apoptosis, while its pathological significance in TBI has not been reported. Herein, we discovered for the first time that ZIPK expression was markedly elevated in neurons after TBI and that ZIPK caused massive neuronal apoptosis in peri-contusional brain regions. *Zipk* haploinsufficiency antagonized neuronal cell death and reversed several typical neuropathological changes induced by TBI. Mechanistically, we found that ZIPK affected neuronal viability by modulating death effector domain-containing DNA binding protein (DEDD) and caspase-3 pathway. Specifically, ZIPK could bind to and phosphorylate DEDD at the S9 residue, thus enhancing the stability of DEDD, and leading to the activation of caspase-3-mediated apoptotic cascade in neurons. The rescue of neuronal loss by ZIPK downregulation effectively alleviated TBI-induced behavioral deficits by preserving motor and cognitive abilities in vivo, supporting the decisive role of ZIPK dysregulation in TBI-associated neuronal dysfunctions by modulating neuronal survival. Furthermore, pharmacological suppression of ZIPK activity by a specific inhibitor prior to TBI protected neurons from brain injury-induced cell death and neuronal degeneration in vitro and in vivo by preventing DEDD upregulation and caspase-3 activation. In conclusion, our data reveal the essential contribution of ZIPK to TBI-induced neuronal cell death through the DEDD/caspase-3 cascade, and suggest the potential of targeting ZIPK as an effective strategy for treating TBI-related neuropathologies.

## Introduction

Traumatic brain injury (TBI) is recognized as a leading cause of disability and death among adults worldwide. The estimated global incidence of TBI is ~56 million cases per year [1]. The high incidence and prevalence of TBI relative to other neurological diseases have made it a major public health burden. TBI involves heterogeneous neuropathological changes at molecular, cellular, and systemic levels that depend on the severity, site, and duration of the injury and interventions [2]. In general, TBI causes primary and secondary injuries, which are initiated by different mechanisms and lead to short-term and long-term neuropathological consequences [3]. Neuronal cell death following TBI results in structural and functional degeneration in both acute and chronic phases, and is considered a driving force for the development of various neuropsychiatric and neurodegenerative disorders [4]. Several types of cell death programs have been reported in the pathogenesis of TBI-related neuronal damage, including apoptosis, necroptosis, and ferroptosis [5–7]. Neuronal cell death not only leads to irreversible tissue damage in the injury area but also negatively affects cells in distal regions through the generation of toxic components, further causing extensive and lasting secondary injuries [4]. Limiting neuronal cell death has been shown to exert protection on preclinical TBI models by reducing cellular damage. Moreover, several anti-apoptotic compounds are undergoing clinical trials for TBI management, albeit with limited progress [4]. However, the upstream regulators of TBI-induced neuronal cell death remain elusive, and the molecular pathways mediating the development of neurobehavioral deficits have not been fully defined.

Zipper-interacting protein kinase (ZIPK), a member of the death-associated protein kinase (DAPK) family, is a serine/threonine (Ser/Thr) protein kinase with important roles in regulating cellular apoptosis and smooth muscle contraction [8]. ZIPK is ubiquitously expressed in different tissues including the brain, and dysregulated ZIPK expression has been shown to be associated with ischemia-induced brain damage and Alzheimer’s disease (AD) [9, 10]. For example, ZIPK participates in regulating blood–brain barrier (BBB) integrity by affecting the permeability of endothelium in ischemia–reperfusion mouse model. Endothelial ZIPK ablation decreases the brain infarct volume and BBB disruption, and improves neuronal functions after ischemia–reperfusion injury [9]. Besides, ZIPK inhibition rescues endothelial senescence through the transcriptional modulation of genes related to energy metabolism and cell survival [11]. An AD patients-based study analyzed a panel of apoptosis-related proteins, and reported higher levels of ZIPK along with other pro-apoptotic proteins in the cortex of AD patients than in age-matched controls [10]. These studies highlight the crucial role of ZIPK dysregulation in the pathogenesis of brain aging and neurological diseases such as ischemia and AD. TBI is a common risk factor for neurodegenerative disorders [12], while whether ZIPK is dysregulated in TBI has not been investigated. Furthermore, the function of ZIPK in the neuropathology of TBI, and specifically in the regulation of neuronal cell death, is incompletely studied.

In this study, we used cellular and animal TBI models to investigate the role of ZIPK in TBI-induced neuronal cell death and underlying mechanisms. We demonstrated that neural injury upregulates the expression of ZIPK, leading to the activation of death effector domain-containing protein (DEDD) and capsapse-3-mediated apoptosis pathway in neurons by phosphorylating the S9 residue of DEDD. ZIPK upregulation following brain injury exacerbates neuronal loss, synaptic impairment, and BBB damage in vivo, ultimately causing neurological impairment and neurocognitive dysfunction. *Zipk* haploinsufficiency or pharmacological inhibition of ZIPK activity efficiently counteracts neuronal cell death and attenuates behavioral deficits in mice, thus providing a potential therapeutic strategy for TBI.

## Materials and methods

### Chemicals and reagents

The details of antibodies used in the study are provided in Table S1. The ZIPK inhibitor was described previously and was purchased from MCE (Shanghai, China) [13]. Cycloheximide (CHX) was purchased from Cell Signaling Technology (Danvers, MA, USA) to inhibit protein synthesis. Hydrogen peroxide (H_2_O_2_) was bought from Ganshanhu (Nanchang, China). Hoechst 33342, Evans blue dye, formamide, and paraformaldehyde powder were obtained from Sangon Biotech (Shanghai, China). Protein A/G plus-agarose was provided by Santa Cruz (Dallas, TX, USA). The protease inhibitor cocktail and phosphatase inhibitor were purchased from Topscience (Shanghai, China). Cytosine-β-d-arabinofuranoside (AraC) was provided by Sigma-Aldrich (Darmstadt, Germany). Turbofect transfection reagent was bought from Thermo Fisher Scientific (Waltham, MA, USA). Tribromoethanol was provided by Sigma-Aldrich. FreeZol reagent was purchased from Vazyme (Nanjing, China). Anti-fading medium was supplied by SouthernBiotech (Birmingham, AL, USA). ZIPK and DEDD constructs were prepared by Dobiotech (Fuzhou, China) and Fenghui Biotech (Changsha, China), respectively. Small interfering RNAs (siRNAs) sequences for *ZIPK* and *DEDD* were synthesized by Hanbio (Shanghai, China), and the specific sequence information is shown in Table S2.

### Animals

Male wild-type (WT) and *Zipk* haploinsufficiency (*Zipk*^+/−^) mice (all in C57BL/6 background) aged 10–12 weeks were used in the present study. The procedure for generating *Zipk*^+/−^ mice via the CRIPSR/Cas9 is shown in Fig. S1A. In brief, a CRISPR/Cas9 system targeting the primary RNA transcript of *Zipk* (ENSMUST00000178422.8) was constructed and microinjected into the fertilized eggs of C57BL/6 mice to get the F0 mice. After confirming the genotypes of F0 mice, the positive F0 mice were mated with WT C57BL/6 mice to obtain F1 generation mice. *Zipk*^+/−^ mice were selected for further breeding and TBI modeling. The sequencing and representative genotyping data are shown in Fig. S1A, B. All mice were maintained at the animal center of Fujian Medical University, on a 12-h light/dark cycle and provided continuous access to food and water. The mouse experiments were approved by the Animal Welfare and Ethics Committee of Fujian Medical University (IACUC FJMU2018-053) and were performed in accordance with institutional guidelines. The sample size was estimated by experience and mice were randomly assigned to each group based on the genotypes.

### TBI mouse model

We applied the controlled cortical impact (CCI) model to induce TBI in mice. The procedure was performed according to previous publications, with minor modifications [14, 15]. Briefly, each mouse was anesthetized with isoflurane and fixed on a stereotaxic frame. The skull surface was exposed, and a 4-mm diameter craniotomy was made in the right parietal bone at −2 mm anteroposterior and −2.5 mm mediolateral from bregma. Afterward, the impact point was aligned perpendicular to the impactor, and the mouse was subjected to CCI using a brain and spinal cord impactor equipped with a flat 3-mm metal tip, which was provided by RWD Life Science (Shenzhen, China). The parameters for CCI were as follows: 3.5 m/s velocity, 0.2 ms dwell time, 2 mm depth. The sham mice were subjected to the same procedure, except for the final impact on the head. The incision was then sutured, and the mouse was placed back to the home cage with a heating pad for recovery. For HS38 treatment in vivo, mice were administrated HS38 (10 mg/kg body weight) or vehicle by intraperitoneal injection 30 min prior to TBI modeling.

### Cell culture and transfection

Human embryonic kidney 293 T (HEK293T) cells were obtained from the Cell Bank/Stem Cell Core Facility (Shanghai, China), and was maintained in Dulbecco’s modified Eagle’s medium (DMEM, Gibco, New York, NY, USA) supplemented with 10% fetal bovine serum (FBS, PAN-biotech, Aidenbach, Germany). The human neuroblastoma cell line SH-SY5Y was purchased from Procell (Wuhan, China) and cultured in MEM/F12 containing 15% FBS (Procell). Plasmids or siRNAs transfection were performed following the manufacturer’s guide. Mouse primary cortical neurons were isolated and cultured as described previously [16]. The isolated neurons were seeded in poly-D-lysine coated 6-well or 24-well plates in a neurobasal medium containing 2% B-27 and GlutaMAX supplement (Gibco). AraC (10 μM) was added at 3 days in vitro to obtain neuron-enriched cultures. Half of the medium was replaced every 2 days. All media contained 100 U/mL penicillin and 100 µg/mL streptomycin (Gibco). The cells were maintained at 37 °C under 5% CO_2_. Primary neurons were used for experiments at seven or 8 days in vitro.

### In vitro TBI model

We employed two cellular models to mimic TBI-induced damage in vitro. For the first model, scratches were made manually with pipette tips to recreate pathological features of penetrating TBI, with minor modifications. Briefly, a cross wound was made in culture well with a 200 μL tip in 6-well plates when cells reached 60-80% confluence [5]. For the second model, to induce oxidative damage, cells were cultured in a fresh cell medium containing 50 or 100 μM H_2_O_2_ for 24 h [7]. Samples were collected at indicated time points for further analysis. For in vitro HS38 treatment, the compound was added to cells at a final concentration of 20 μM 2 h before scratch injury or H_2_O_2_ treatment.

### Quantitative real-time PCR

Total RNA was extracted from various samples according to the manufacturer’s instructions. Quantitative real-time PCR (qRT-PCR) was performed based on our previous protocol [17]. In brief, cDNA was synthesized from total RNA using the HiScript® III RT SuperMix (Vazyme) following the manual. The resulting cDNA was then amplified by qRT-PCR via the ChamQ Universal SYBR qPCR Master Mix (Vazyme) through the QuantStudio Real-time PCR System (Applied BioSystems, Waltham, MA, USA) under the following temperature cycling conditions: 95 °C for 3 min, then 40 cycles of 95 °C for 20 s, 60 °C for 30 s, and 72 °C for 30 s were performed. Data were analyzed using the 2^−ΔΔCt^ method, and *ACTB* was used as an endogenous control [18]. The primers used are shown in Table S3.

### Immunoblotting analysis

Total proteins were extracted from cells or brain tissues with radioimmunoprecipitation assay (RIPA) buffer. The protein concentration was determined with a BCA protein assay kit (Beyotime, Shanghai, China). Protein samples (15–20 μg) were separated by sodium dodecyl sulfate-polyacrylamide gel electrophoresis (SDS-PAGE) and transferred to polyvinylidene difluoride membranes (Merck, Darmstadt, Germany) via semi-dry transfer (Bio-Rad, Hercules, CA, USA). Membranes were then blocked with 5% bovine serum albumin or non-fat milk in TBST for 1 h at room temperature and subsequently incubated with primary antibodies overnight at 4 °C. After washing three times with TBST, HRP-conjugated secondary antibodies were added to membranes, and incubated for 1 h at room temperature. After being rinsed in TBST three times, all membranes were developed using a Chemidoc imaging system (Bio-Rad). Protein levels were quantified by densitometry analysis using β-actin as a loading control in ImageJ software (version 1.51j8), and the protein level in each group was normalized to that in the control group for further comparison.

### Co-immunoprecipitation and in vitro kinase assay

Co-immunoprecipitation (Co-IP) and in vitro kinase analyses were performed as described previously [19]. Total protein was extracted from brain tissues or cells with NP-40 lysis buffer, and the extracted proteins were incubated with primary antibodies at 4 °C for 2 h. The immune complexes were pulled down with protein A/G plus-agarose at 4 °C for 1 h. The agarose was collected and washed, and proteins were eluted by boiling in 2× loading buffer before immunoblotting analysis. For in vitro kinase assay, recombinant ZIPK (Thermo Fisher Scientific) was incubated with His-DEDD or GST-MLC purified from bacterial expression in kinase reaction buffer at room temperature for up to 30 min [20]. Samples were subjected to ^32^P-autoradiography as described previously [19]. Samples after the kinase assay were also separated in SDS-PAGE, and the DEDD bands were collected for mass spectrometry (Biotech-pack, Beijing, China) or incubated with an anti-pan-phospho-Ser/Thr antibody (Abcam, Cambridge, UK).

### Immunostaining assay

Immunostaining measurement was conducted as described in previous studies [21, 22]. Mice were anesthetized by intraperitoneal injection of 1.25% tribromoethanol (0.2 mL/10 g body weight), and brains were immediately isolated and fixed in 4% paraformaldehyde (w/v in PBS) overnight. Afterward, brain tissues were sequentially dehydrated in ethanol solutions, and embedded in paraffin. Coronal sections (5 μm) were cut serially using a microtome from Leica (Shanghai, China). After deparaffinization, hydration, and antigen retrieval, sections were blocked in goat serum (Solarbio, Beijing, China) for 1 h at room temperature. Samples were incubated with primary antibodies at 4 °C overnight and washed with PBS containing 0.1% Tween-20 three times. All samples were then incubated with Alexa 488- or 546-conjugated secondary antibodies (Thermo Fisher Scientific) and Hoechst 33342 (1:2000) for 1 h at room temperature. Images were acquired with a fluorescence microscope (Axio Imager 2, Zeiss, Oberkochen, Germany), and signals were analyzed using ImageJ software. The expression of a certain protein was presented as the relative level to that of the control.

### TUNEL assay

TUNEL assay was performed via an in situ cell death detection kit (TMR Red) obtained from Sigma-Aldrich. Cell samples or brain sections were processed following the manufacturer’s instructions, and nuclei were visualized by Hoechst 33342. Images were acquired in a fluorescence microscope (Axio Imager 2, Zeiss), and the number of TUNEL-positive cells in each sample was quantified with ImageJ.

### Nissl staining

Neuronal damage was detected according to the instructions of Nissl staining solution (Beyotime). Paraffin mouse brain sections were deparaffinized and hydrated according to the procedures of immunostaining. Nissl staining solution was added, and sections were incubated at 37 °C for 10 min in the dark, washed twice with ddH_2_O, and incubated with 95% ethanol and xylene for 5 min each. After drying, the sections were sealed with neutral resin (Solarbio). All samples were imaged with a light microscope (Primo Star, Zeiss). Cells with large, full soma and round nuclei are recognized as intact neuron, while cells showing shrunken morphology and condensed nuclear staining are defined as disorganized neuron [23].

### Evans blue staining

BBB damage was evaluated by measuring Evans blue dye uptake [24, 25]. At 48 h after TBI, 2% Evans blue dye was injected through the tail vein of mice (2 mL/kg) 2 h before brain isolation. Mice were anesthetized and perfused with pre-cooled PBS solution. Brain tissues from the injured hemisphere were harvested and weighed, cut into pieces, and placed in a test tube containing 1 mL formamide. All samples were incubated at 60 °C for 24 h and centrifuged at 14,000 rpm for 30 min to collect the supernatants. Evans blue standard solutions were prepared. The absorbance values of the sample supernatants and standard solutions were measured at 620 nm using a microplate reader (Thermo Fisher Scientific), and the content of Evans blue in each sample was determined based on the standard curve and normalized to the brain weight.

### Modified neurological severity score (mNSS)

mNSS was determined as previously described to evaluate the neurological deficits of mice [26]. Neurological deficits were evaluated 1, 2, 3, and 7 days after TBI. Scores ranged from 0 to 12, with higher scores indicating more severe neurologic deficits in the mice (Table S4). The data were evaluated in a blinded manner.

### Open-field test (OFT)

OFT is used to measure the exploratory locomotor and anxiety-like behavior of our mouse models [27]. Mice were brought to the test room at least 30 min before the test. During the measurement, each mouse was placed in the center of an open field (40 cm × 40 cm) and allowed to explore for 15 min. The movement was recorded and the trajectory of the last 10 min of the test was analyzed to obtain the time and distance traveled in central area and the number of entries into the central area.

### Morris water maze (MWM) test

The spatial learning and memory abilities of mice were measured using the MWM test. The method was described in our previous study [28]. In brief, all mice were transferred to the test room 30 min before the start of the experiment. Titanium dioxide was used to enhance the contrast, and the water temperature was maintained at 25 ± 1 °C. Mice were first subjected to a visible platform test (day 1, platform 1 cm above water) and hidden platform tests (days 2 to 5, platform 1 cm below water) in a water maze equipped with a camera, with five trials per day (inter-trial intervals of ~20 min). The cutoff time for each trial was set to 60 s. If the mouse reached the platform within 60 s, it was allowed to remain on the platform for 5 s. Otherwise, the mouse was led to the platform and allowed to stay there for 20 s. The probe trial was performed on day 6, in which the platform was removed and mice were allowed to swim freely in the water for 60 s. Swimming path was recorded and analyzed by the Smart program (version 3.0.06) provided by Panlab Harvard Apparatus (Barcelona, Spain).

### Statistical analysis

GraphPad Prism software (San Diego, CA, USA) was used for data analysis. All data are presented as means ± standard deviation (SD) from at least three independent experiments. The normality of the data was first evaluated via Shapiro–Wilk test. An unpaired two-tailed Student’s *t* test was applied to analyze the difference between two groups, whereas one-way analysis of variance (ANOVA) followed by Tukey’s multiple comparisons test was applied to compare multiple groups. Non-normal distribution data were compared by nonparametric tests. The group size and statistical methods were detailed in figure legends. *P* < 0.05 was considered statistically significant.

## Results

### TBI leads to upregulation of ZIPK expression in neurons

**Fig. 1:**
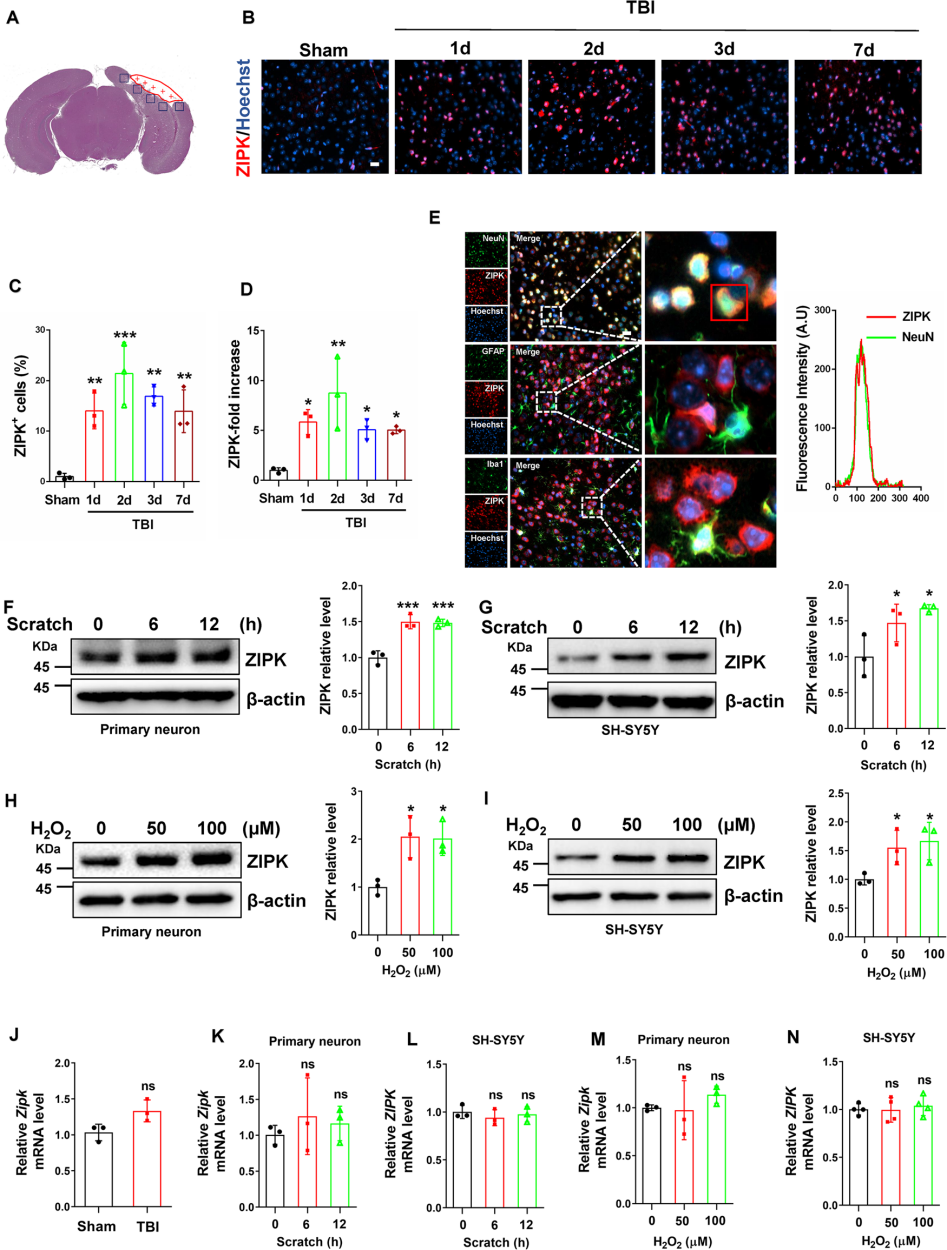
ZIPK expression is increased after TBI, scratch injury, and H_2_O_2_ exposure. **A** Schematic representation of the contusional region (red) and the peri-contusional areas (blue) after TBI. Levels of all pathological markers were detected from the peri-contusional area (blue). **B** Immunofluorescence analysis of ZIPK expression in peri-contusional brain areas of mice from sham and TBI groups at different time points after injury. *n* = 3 mice/group. The scale bar is 20 μm. **C**, **D** Quantification of the ZIPK^+^ cell number and relative ZIPK signal intensity via ImageJ. **E** Representative immunostaining of ZIPK and NeuN (neuronal marker), GFAP (astrocyte marker) or Iba1 (microglial marker) in peri-contusional brain areas. The cells used for colocalization analysis are marked in red. The scale bar is 20 μm. **F**, **G** Representative immunoblots and quantification of ZIPK expression in primary neurons and SH-SY5Y cells after scratch injury **H**, **I** Immunoblot analysis of ZIPK levels in primary neurons and SH-SY5Y cells treated with different concentrations of H_2_O_2_. **J**–**N** qRT-PCR analysis of *ZIPK* mRNA levels in in vivo and in vitro TBI models. *P < 0.05, **P < 0.01, ***P < 0.001, ns, not significant. One-way ANOVA was used for statistics in (**C**–**I**, **K**–**N**). Unpaired two-tailed Student’s *t* test was used in (**J**).

To determine whether ZIPK is involved in the neuropathology of TBI, we evaluated the temporal changes in the expression of ZIPK in peri-contusional regions after CCI-induced brain injury (Fig. 1A). Immunofluorescence revealed a remarkable increase in ZIPK protein level as well as the number of ZIPK-positive cells in peri-contusional brain areas of mice 1 day following CCI. The induction of ZIPK expression in peri-injury sites peaked at 2 days post TBI, and sustained for one week based on our analysis (Fig. 1B–D). We further confirmed that TBI upregulated ZIPK primarily in neurons, as shown by the high colocalization of ZIPK with the neuronal cell marker NeuN, but not with glial cell markers GFAP and Iba1 (Fig. 1E). Having discovered the upregulation of neuronal ZIPK after TBI, we examined whether ZIPK expression was also increased in in vitro TBI models by subjecting neurons to scratch injury or H_2_O_2_-induced oxidative damage. Scratch injury recapitulates the primary mechanical damage in open-head injury, and can trigger various secondary injury mechanisms through the release of harmful factors such as oxygen-free radicals from damaged neurons [5, 29]. Furthermore, the increase in oxygen-free radicals is an important pathological change in TBI [7, 29]. Consistent with the in vivo data, ZIPK protein levels in primary neurons and SH-SY5Y cells were markedly increased after the scratch injury or H_2_O_2_ exposure (Fig. 1F–I). Thus, the findings of animal and cellular studies corroborate the dysregulation of neuronal ZIPK expression after TBI. However, the *ZIPK* mRNA level was not significantly altered in our TBI models (Fig. 1J–N). Taken together, these results demonstrate that TBI leads to ZIPK upregulation in neurons.

### ZIPK dysregulation mediates TBI-induced neuronal cell death

**Fig. 2:**
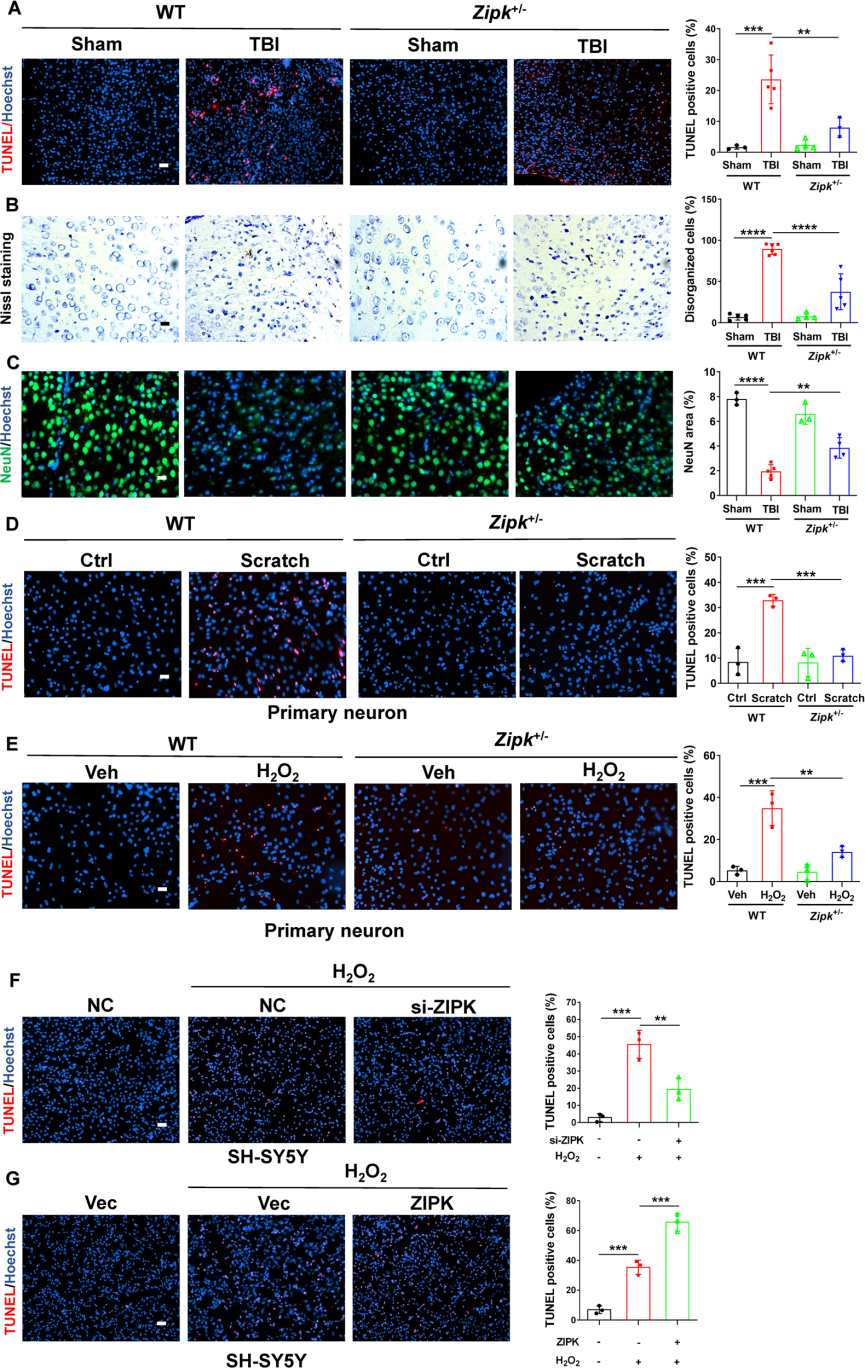
ZIPK modulates TBI-induced neuronal cell death in vivo and in vitro. **A** TUNEL assay was used to analyze neuronal death in the peri-injury site in WT and *Zipk*^+/−^ mice. The scale bar is 50 μm. **B** Nissl staining was used to compare the percentage of disorganized neurons at peri-injury regions between WT and *Zipk*^+/−^ mice after TBI. The scale bar is 20 μm. **C** NeuN immunostaining was used to measure the area of cortical neurons in peri-injury regions. The scale bar is 20 μm. **D**, **E** TUNEL assay showing the death of WT or *Zipk*^+/−^ primary neurons treated with scratch (**D**) or H_2_O_2_ (**E**). The scale bar is 50 μm. **F**, **G** TUNEL assay showing the death of SH-SY5Y cells with ZIPK knockdown (**F**) or HA-ZIPK overexpression (**G**) in the presence of H_2_O_2_ treatment. The scale bar is 100 μm. Blue staining indicates cell nuclei and magenta staining represents TUNEL signal. *n* = 3–6 mice/group for (**A**–**E**). NC negative control. **P < 0.01, ***P < 0.001, ****P < 0.0001. One-way ANOVA was used for comparison.

Neuronal cell death is a pivotal pathological feature of TBI and may occur in acute and chronic phases of brain injury due to primary mechanical damage or secondary neurochemical changes. ZIPK has been proven to be closely involved in regulating cell apoptosis. Besides, a previous study reported the upregulation of ZIPK and several pro-apoptotic proteins in the cortices of AD patients [10], implicating a potential role of ZIPK in regulating neuronal integrity in neurological disorders. To elucidate whether ZIPK is a key regulator of TBI-induced neuronal death, we first generated a *Zipk*^+/−^ mouse model using the CRISPR/Cas9 technique (Fig. S1A, B). While *Zipk* knockout is embryonic lethal [9, 30], systemic *Zipk* haploinsufficiency had no obvious effect on brain morphology and body size in either the postnatal or adult stage (Fig. S1C–F). The expression of ZIPK was reduced by about 50% in *Zipk*^+/−^ mouse brain and primary neurons compared with those in WT littermates (Fig. S1G–I). Moreover, the upregulation of ZIPK in peri-injury sites of WT-TBI mice was significantly mitigated in *Zipk*^+/−^-TBI mice (Fig. S2). We first performed TUNEL assay to detect neuronal cell death in WT and *Zipk*^+/−^ mice after TBI. The significant increase in TUNEL-positive signals in peri-contusional areas of WT-TBI mice indicated the occurrence of massive cell death in the brain following TBI. However, this increase was ameliorated in *Zipk*^+/−^-TBI mice (Fig. 2A). The protection was further supported by results of Nissl staining, showing that the number of damaged neurons in peri-injury regions was much lower in *Zipk*^+/−^-TBI group than in WT-TBI group (Fig. 2B). In line with the TUNEL assay and Nissl staining, NeuN immunostaining also showed about 70% neuronal loss in brains of WT-TBI mice (Fig. 2C). However, *Zipk* haploinsufficiency attenuated TBI-induced neuronal loss as exhibited by an increase in the number of NeuN-positive cells. The in vivo data clearly confirmed the essential role of ZIPK in mediating TBI-induced neuronal loss. To further clarify the effect of ZIPK on neuronal cell death, primary neurons from WT and *Zipk*^+/−^ mice were subjected to scratch or H_2_O_2_ treatment, and cell death was quantified using TUNEL assay. A partial deletion of ZIPK also considerably protected neurons against scratch or H_2_O_2_-induced cell death (Fig. 2D, E). Furthermore, we transfected SH-SY5Y cells with si-ZIPK or HA-ZIPK constructs to silence or increase ZIPK expression, respectively. ZIPK silencing by si-ZIPK treatment inhibited H_2_O_2_-induced cell death (Fig. 2F), whereas ZIPK overexpression further aggravated oxidative damage-induced cell apoptosis (Fig. 2G). Combined, the above results show that ZIPK upregulation contributes to TBI-induced neuronal cell death.

### ZIPK induces neuronal cell death by regulating the DEDD/caspase-3 pathway

**Fig. 3:**
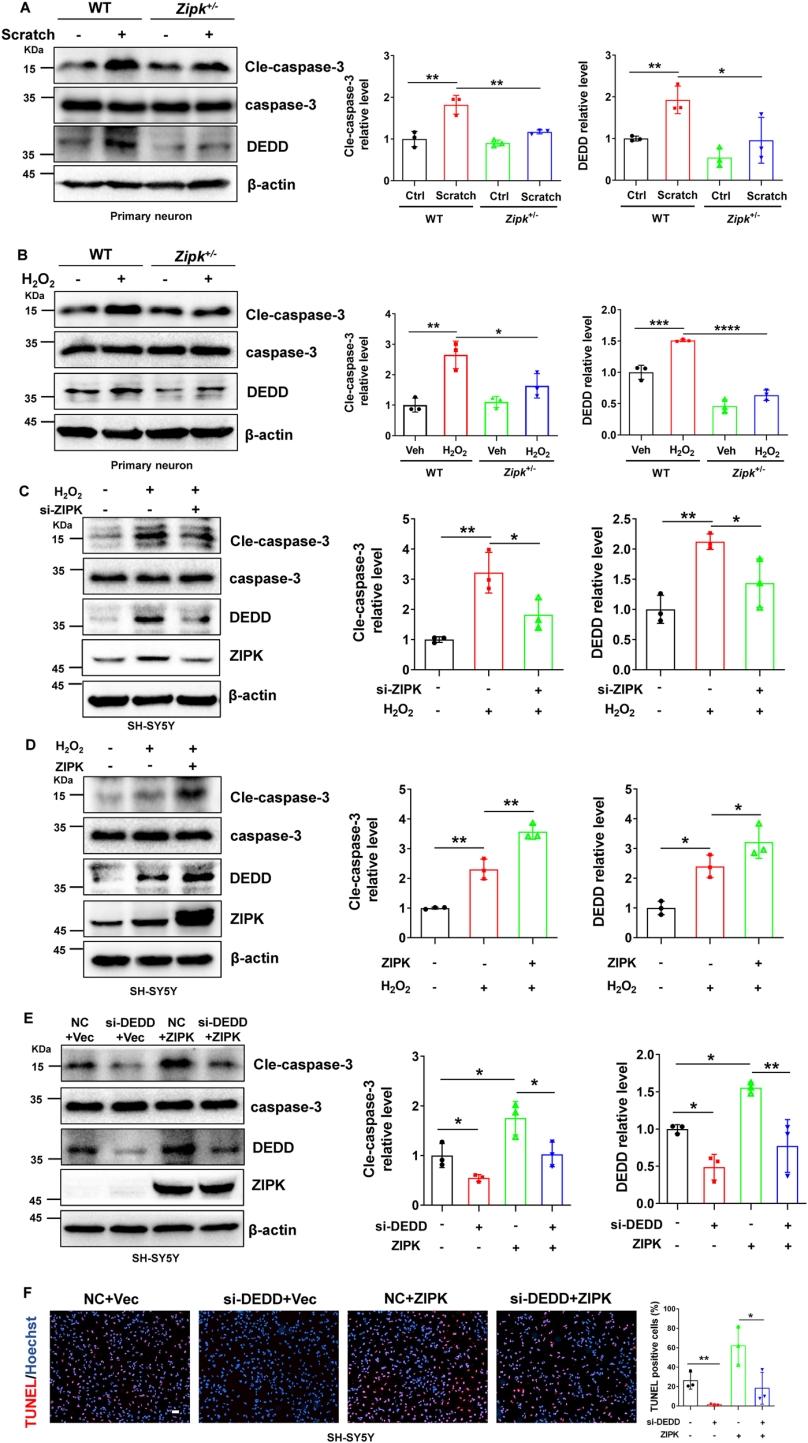
ZIPK induces neuronal cell death by regulating the DEDD/caspase-3 pathway. **A**, **B** Representative immunoblots and quantification of DEDD and cle-caspase-3 levels in WT and *Zipk*^+/−^ primary neurons treated with scratch (**A**) or H_2_O_2_ (**B**). **C**, **D** SH-SY5Y cells were transfected with si-ZIPK (**C**) or HA-ZIPK (**D**), and treated with or without H_2_O_2_, after which immunoblotting analysis of DEDD and cle-caspase-3 was performed. **E** Immunoblot images of cle-caspase-3, DEDD, and ZIPK in SH-SY5Y cells transfected with HA-ZIPK or si-DEDD alone or both. The relative levels of cle-caspase-3 and DEDD were evaluated. **F** TUNEL assay showing the death of SH-SY5Y cells transfected with HA-ZIPK or si-DEDD alone or both. The scale bar is 100 μm. NC negative control. *P < 0.05, **P < 0.01, ***P < 0.001, ****P < 0.0001. One-way ANOVA was used for analysis.

To better understand the molecular pathways underlying ZIPK dysregulation and neuronal cell death in TBI, we evaluated levels of several key molecules responsible for cell apoptosis in cellular TBI models. The hyperactivation of caspases has been noted in both ZIPK-induced apoptosis and TBI-induced cell death [31, 32]. The activity of caspase-3, a major executor of cell apoptosis, is tightly modulated by DEDD. DEDD participates in the regulation of cell death via a caspase-3-dependent pathway [33, 34]. Besides, the expression of DEDD is elevated in the cortices of AD patients [35]. Moreover, several potential ZIPK phosphorylation motifs exist in DEDD protein. This evidence suggests that DEDD may be a link between ZIPK and TBI-induced neuronal death. We thus focused on DEDD and caspase-3 as downstream molecules of ZIPK in cellular TBI models. We observed that the protein levels of cleaved-caspase-3 (cle-caspase-3) and DEDD were increased in WT neurons after scratch injury or H_2_O_2_ exposure (Fig. 3A, B). However, this increase was reversed in *Zipk*^+/−^ neurons subjected to scratch or H_2_O_2_ (Fig. 3A, B). In addition, SH-SY5Y cells transfected with si-ZIPK or HA-ZIPK were treated with H_2_O_2_, and we found that ZIPK knockdown prevented H_2_O_2_-induced increase in cle-caspase-3 and DEDD expression (Fig. 3C), whereas ZIPK overexpression promoted H_2_O_2_-induced upregulation of DEDD and cle-caspase-3 (Fig. 3D). These results substantiate that ZIPK modulates the DEDD/caspase-3 pathway in TBI.

To explore whether DEDD is the key molecule in ZIPK-mediated cell death, we then examined whether silencing DEDD expression with si-DEDD affects H_2_O_2_-triggered cell death in SH-SY5Y cells. We found that H_2_O_2_-induced cle-caspase-3 formation was diminished after DEDD knockdown (Fig. 3E). More importantly, H_2_O_2_-induced aggravation of caspase-3 activation in ZIPK-overexpressing SH-SY5Y cells was suppressed after DEDD knockdown (Fig. 3E). Notably, DEDD knockdown not only protected against H_2_O_2_-induced SH-SY5Y cell death, but also efficiently restrained the ZIPK overexpression-induced apoptosis in the TUNEL analysis (Fig. 3F), which was consistent with the immunoblotting data. These results together indicate that ZIPK-mediated cell death depends on DEDD.

### ZIPK regulates the protein expression of DEDD by affecting its stability

**Fig. 4:**
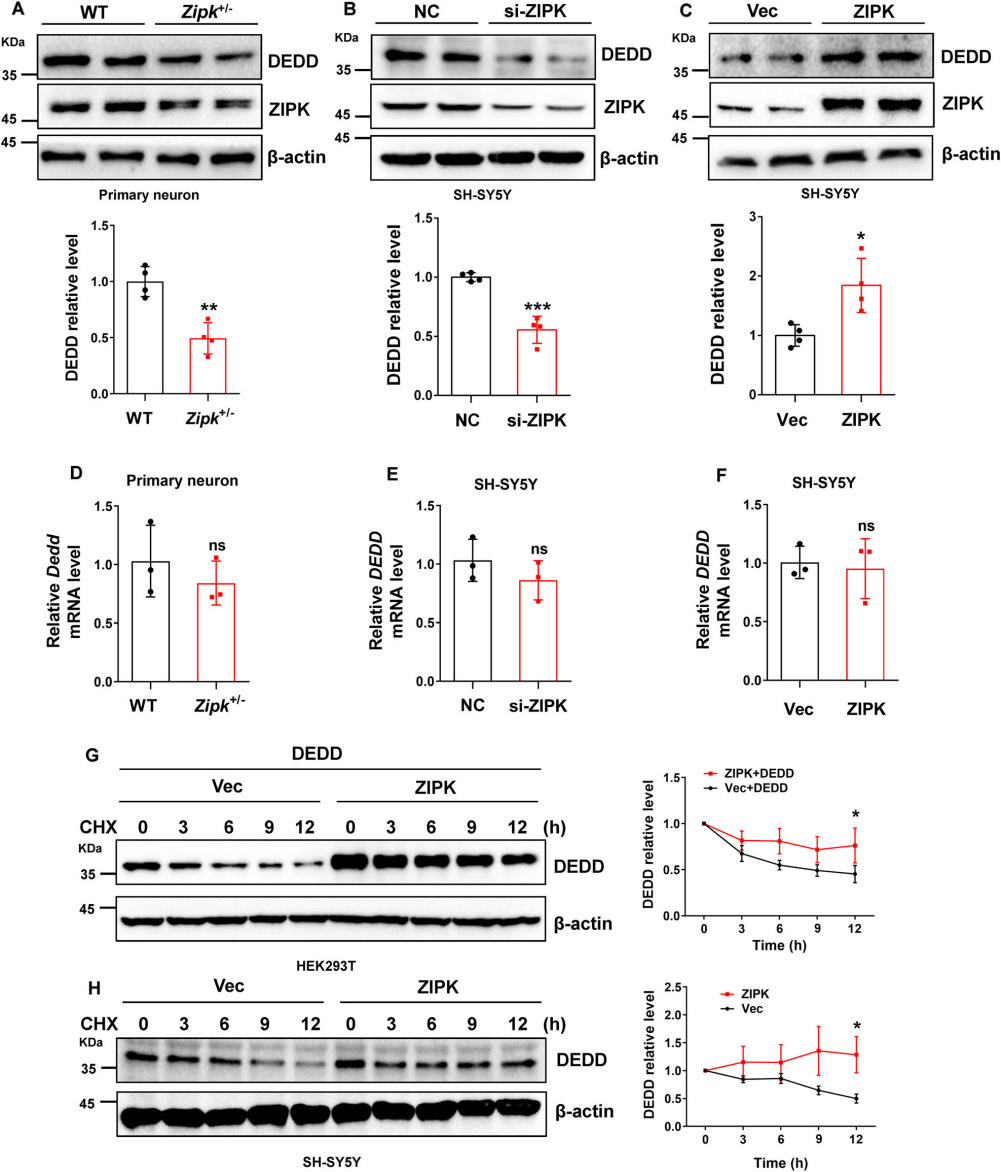
ZIPK regulates the expression of DEDD by affecting its stability. **A**–**C** Immunoblot analysis of DEDD expression in primary neurons from WT and *Zipk*^+/−^ mice (**A**), SH-SY5Y cells with ZIPK knockdown (**B**) or overexpression (**C**). **D**–**F** *DEDD* mRNA levels were analyzed by qRT-PCR in WT and *Zipk*^+/−^ primary neurons (**D**) and SH-SY5Y cells with ZIPK knockdown (**E**) or overexpression (**F**). **G** HEK293T cells were transiently transfected with HA-vector or HA-ZIPK and Flag-DEDD constructs and subjected to a CHX assay. Representative immunoblot showing DEDD levels at different time points after CHX addition was shown. **H** SH-SY5Y cells were transiently transfected with HA-vector or HA-ZIPK constructs and then treated with CHX for the indicated duration, followed by immunoblot quantification of DEDD protein levels. NC, negative control. *P < 0.05, **P < 0.01, ***P < 0.001, ns, not significant. Unpaired two-tailed Student’s *t* test was used for analysis.

Having identified DEDD as a downstream target of ZIPK in modulating TBI-induced neuronal cell death, we next aimed to investigate whether and how ZIPK regulates DEDD expression. First, we observed that DEDD expression was decreased by about 50% in *Zipk*^+/−^ primary neurons compared with WT neurons (Fig. 4A). Like in primary neurons, endogenous DEDD expression was reduced by about 50% in SH-SY5Y cells with ZIPK knockdown (Fig. 4B). In contrast, there was a noticeable increase in the protein level of DEDD after ZIPK was overexpressed in SH-SY5Y cells (Fig. 4C). Nevertheless, there was no significant change in the *DEDD* mRNA level, regardless of the ZIPK expression level in these cells (Fig. 4D–F). Moreover, *DEDD* mRNA levels remained constant in our mouse and cellular TBI models (Fig. S3).

Since ZIPK does not affect *DEDD* transcription, we wanted to study whether ZIPK affects DEDD protein levels through post-transcriptional regulation. We assessed the protein stability of DEDD in HEK293T cells following ZIPK and DEDD overexpression via a CHX chase assay, in which the protein synthesis was suppressed by CHX treatment. The results showed that ZIPK overexpression decelerated the degradation of DEDD (Fig. 4G), suggesting the stabilization of DEDD when ZIPK is upregulated. Similarly, the half-life of endogenous DEDD in SH-SY5Y was significantly prolonged after ZIPK overexpression (Fig. 4H). These results indicate that ZIPK upregulation leads to DEDD accumulation in TBI models by increasing DEDD protein stability.

### ZIPK directly binds to and phosphorylates DEDD, thereby affecting its stability

**Fig. 5:**
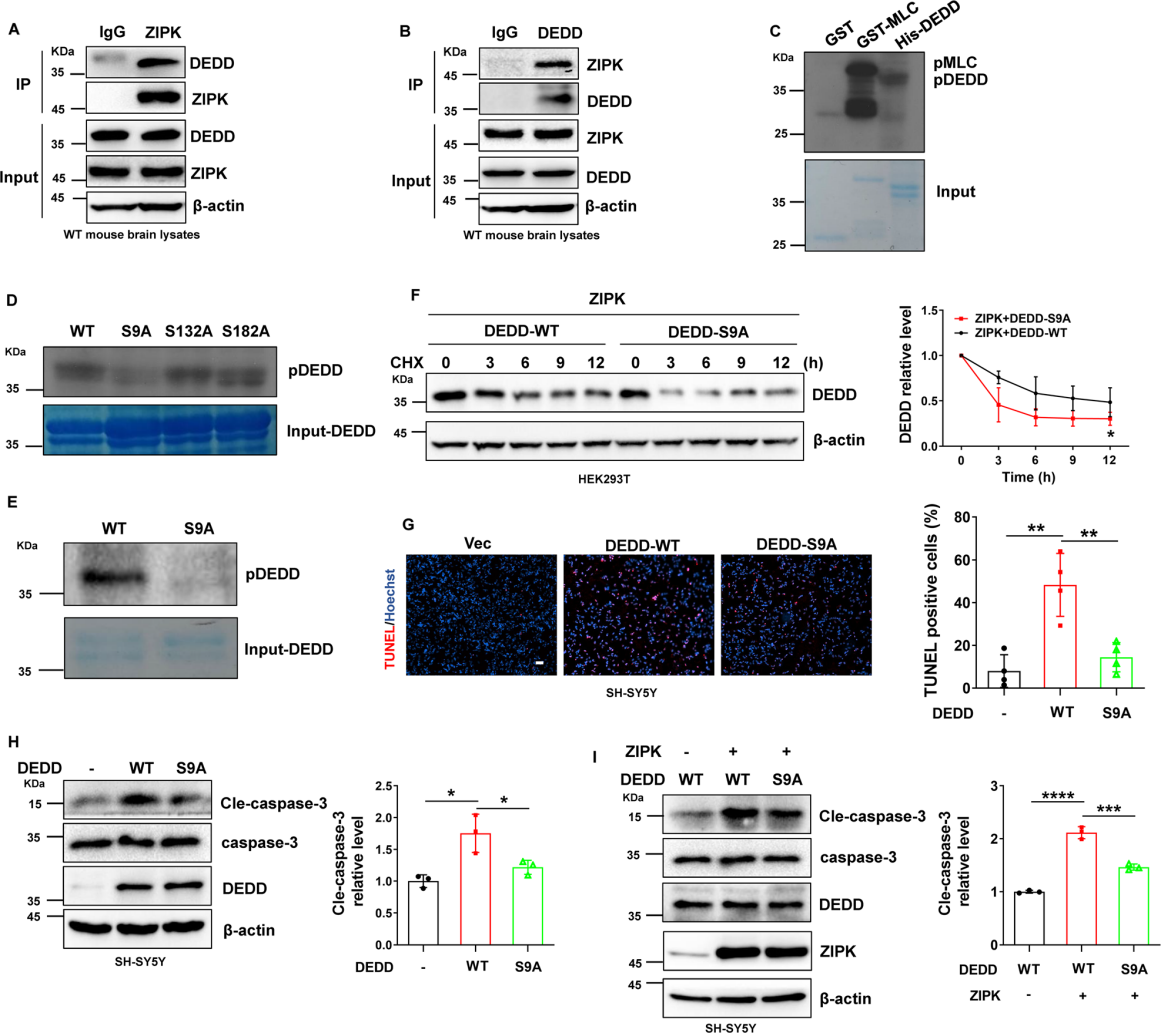
ZIPK directly binds to and phosphorylates DEDD, thereby affecting its stability. **A**, **B** Endogenous binding of ZIPK and DEDD in mouse brain tissues in the Co-IP assay using anti-ZIPK (**A**) or -DEDD (**B**) antibody, respectively. **C** Representative autoradiography data from the in vitro kinase assay using ^32^P-ATP and purified recombinant proteins. GST-MLC and GST were positive and negative controls, respectively. **D** ^32^P-autoradiography data for WT or alanine mutants of DEDD in in vitro kinase assay. **E** Phosphorylation of DEDD-WT or S9A mutant detected by an anti-pan-phospho-antibody. **F** Immunoblot analysis of DEDD-WT and S9A mutant levels in HEK293T cells transiently transfected with Flag-DEDD-WT or Flag-DEDD-S9A and HA-ZIPK constructs in the CHX assay. **G**, **H** TUNEL assay (**G**) and immunoblot analysis of cle-caspase-3 (**H**) in SH-SY5Y cells expressing Flag-DEDD-WT or Flag-DEDD-S9A and treated with H_2_O_2_. The scale bar is 100 μm. **I** Expression levels of cle-caspase-3 in SH-SY5Y cells after co-transfection of HA-ZIPK with Flag-DEDD-WT or Flag-DEDD-S9A and treated with H_2_O_2_. *P < 0.05, **P < 0.01, ***P < 0.001, ****P < 0.0001. Unpaired two-tailed Student’s *t* test was used in (**F**). One-way ANOVA was used in (**G**–**I**).

Our results revealed that ZIPK-mediated increase in DEDD protein content is an important contributor to neuronal cell death and that ZIPK upregulation leads to increased DEDD protein stability. We further studied potential mechanisms by which ZIPK upregulates DEDD protein stability in vitro. DEDD protein contains several consensus ZIPK phosphorylation motifs, making it a potential substrate of ZIPK [9, 11, 36]. We thus speculated that ZIPK may alter the protein stability of DEDD by directly phosphorylating DEDD. Therefore, we first analyzed whether there is an interaction between ZIPK and DEDD. Co-IP assays using anti-ZIPK or -DEDD antibodies were performed on mouse brain lysates. We could detect a high abundance of DEDD in samples precipitated with anti-ZIPK antibody (Fig. 5A), indicating an interaction between these two proteins in the brain. The interaction was corroborated by the reciprocal immunoprecipitation using anti-DEDD antibody (Fig. 5B). We further confirmed the binding in HEK293T cells and in SH-SY5Y cells through the Co-IP assay (Fig. S4). To explore whether ZIPK can directly phosphorylate DEDD, we purified recombinant His-DEDD protein and conducted in vitro kinase assay using GST-ZIPK and ^32^P-ATP. GST and GST-MLC were negative and positive controls, respectively. Obvious phosphorylation was observed in GST-MLC and His-DEDD samples (Fig. 5C), and the signal for phospho-DEDD increased in a time-dependent manner (Fig. S5A), indicating a direct phosphorylation of DEDD by ZIPK. Mass spectrometry was used to identify the specific amino acid sites of ZIPK-phosphorylated DEDD. The analysis reported three potential phosphorylation sites in DEDD, namely S9, S132, and S182 (Fig. S5B, C), all of which are in the consensus ZIPK phosphorylation motifs. To determine the exact amino acid residue phosphorylated by ZIPK, we mutated the three putative phosphorylation sites to alanine and conducted ^32^P-autoradiography. We observed that only DEDD-S9A but not the other two mutants showed diminished signal in the kinase assay (Fig. 5D). The phosphorylation site was further corroborated using a pan-phosphorylation antibody (Fig. 5E). The results suggest that the ZIPK might directly phosphorylate DEDD at S9.

Given the influence of ZIPK on DEDD degradation, we wanted to illuminate whether ZIPK regulates the protein stability of DEDD by specifically phosphorylating the S9. We co-transfected HA-ZIPK with Flag-DEDD-WT or Flag-DEDD-S9A into HEK293T cells and performed the CHX assay to compare protein stability. The stability of DEDD-S9A mutant was lower than that of DEDD-WT, as revealed by the accelerated degradation of S9A mutant after CHX addition (Fig. 5F). Through the above data, we conclude that ZIPK upregulates DEDD protein stability likely by phosphorylating its S9 residue.

To further assess the functional relevance of ZIPK-induced DEDD phosphorylation in TBI, we transfected DEDD-WT or DEDD-S9A into SH-SY5Y cells and evaluated whether the S9A mutation abolishes H_2_O_2_-induced cell death and caspase-3 activation. We noticed a significant decline in TUNEL staining signals in DEDD-S9A-transfected cells compared with the DEDD-WT cells (Fig. 5G). Similarly, the S9A mutation normalized the level of cle-caspase-3 induced by DEDD overexpression (Fig. 5H). The suppression of caspase-3 activation by DEDD-S9A mutant was also detectable in SH-SY5Y cells with ZIPK overexpression (Fig. 5I). Taken together, these data indicate that the phosphorylation of DEDD at S9 plays a critical role in ZIPK-induced cell death in TBI.

### ZIPK downregulation alleviates TBI-induced neuropathological changes

**Fig. 6:**
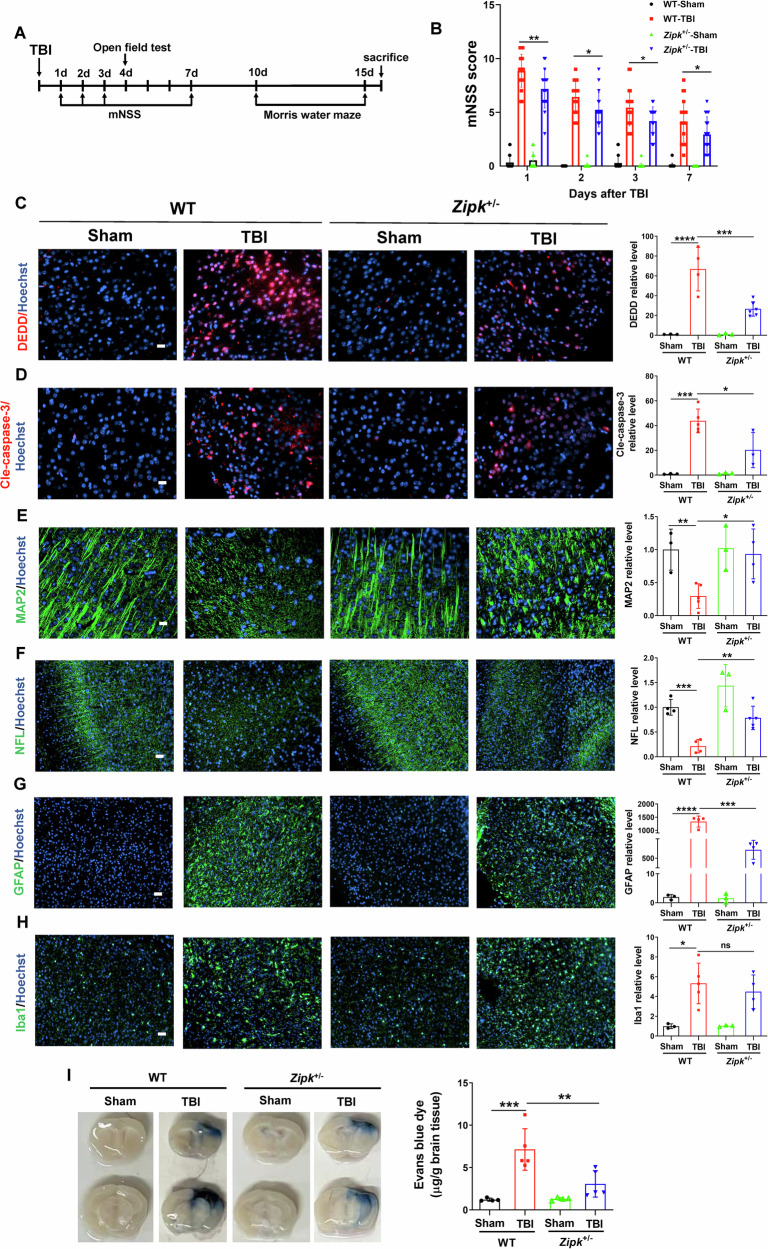
ZIPK downregulation alleviates TBI-induced neuropathological changes. **A** Schematic showing the timeline of the neurobehavioral tests. **B** Neurological function was evaluated by mNSS analysis on days 1, 2, 3, and 7 post-TBI. *n* = 17–21 mice/group. **C**, **D** Representative immunostaining of DEDD (**C**) and cle-caspase-3 (**D**) expression in peri-contusional areas in WT and *Zipk*^+/−^ mice at 16 days after TBI. The scale bar is 20 μm. **E**, **F** Representative immunofluorescence images of MAP2 (scale bar, 20 μm) and NFL (scale bar, 50 μm) in peri-contusional areas in WT and *Zipk*^+/−^ mice at 16 days after TBI. **G**, **H** Analysis of astrocyte or microglia activation by GFAP and Iba1 immunostaining in peri-contusional regions in WT and *Zipk*^+/−^ mice at 16 days after TBI. The scale bar is 50 μm. *n* = 3–6 mice/group for (**C**–**H**). The immunofluorescence signals in (**C**–**H**) were expressed as relative levels to those of WT-Sham. **I** BBB damage in WT and *Zipk*^+/−^ mice was detected by Evans blue staining at 2 days post TBI, *n* = 4–5 mice/group. *P < 0.05, **P < 0.01, ***P < 0.001, ****P < 0.0001. One-way ANOVA test used for analysis.

The beneficial effect of ZIPK knockdown on neuronal cell death in cell and mouse TBI models prompted us to further check whether reducing ZIPK expression protects against neuropathological changes induced by TBI in vivo. The timeline for behavioral tests and pathological examinations is exemplified in Fig. 6A. Modified neurological severity scoring (mNSS) was first performed to determine whether *Zipk* haploinsufficiency promotes the recovery of neurological function after TBI. The mNSS values of WT-TBI mice were significantly higher than those of WT-Sham mice at all tested time points, suggesting impaired neuronal function after CCI. However, mice in *Zipk*^+/−^-TBI group presented lower scores than those in WT-TBI group throughout the test (Fig. 6B), indicating that *Zipk* haploinsufficiency is able to attenuate TBI-induced neurological dysfunction. Importantly, TBI significantly upregulated DEDD and cle-caspase-3 expression in the peri-injury regions in WT mice, and this change was ameliorated in *Zipk* ^+/−^ mice (Fig. 6C, D). These findings reinforced the in vitro data (Fig. 3) and explained the positive effects of *Zipk* haploinsufficiency on neuronal cell death in vivo (Fig. 2). In addition to neuronal loss, TBI-induced brain injury is also characterized by neurite abnormalities, neuroinflammation, and BBB breakdown. Nerve microtubule disassembly is a distinctive neuropathological change induced by TBI, in which mechanical damage disrupts neurite structures, resulting in dendritic and axonal impairments [37–40]. We thus studied the expression of microtubule-associated protein 2 (MAP2) and neurofilament light chain (NFL), markers of dendrites and axons, respectively, to quantify neurite injuries in our models. TBI caused a significant reduction in both MAP2 and NFL signals in the peri-contusional areas in WT-TBI mouse brains. However, *Zipk* haploinsufficiency reversed the decline in MAP2 and NFL caused by TBI (Fig. 6E, F). These data hint that reducing ZIPK expression can mitigate TBI-induced dendritic and axonal degeneration. The activation of astrocytes and microglial cells is one of the reasons for promoting the secondary injury of TBI [41]. Previous research reported that ZIPK can regulate inflammation [42, 43]. We measured the expression of GFAP and Iba1, markers for astrocytes and reactive microglia, respectively, in mouse models. We observed that TBI significantly stimulated GFAP and Iba1 expression in WT mouse brains, indicating the presence of a neuroinflammatory response after injury. There was a significant decrease in the expression of GFAP in *Zipk*^+/−^-TBI mice, but the expression of Iba1 did not change significantly between WT and *Zipk*^+/−^ mice exposed to TBI (Fig. 6G, H). BBB breakdown evoked by TBI was shown by a rapid and extensive increase in the Evans blue content in the brains of WT mice 2 days after TBI. However, the level of Evans blue was significantly reduced in the brains of *Zipk*^+/−^-TBI mice, which suggests that *Zipk* haploinsufficiency is capable of mitigating BBB damage caused by TBI (Fig. 6I). The in vivo data comprehensively validated that ZIPK upregulation results in neurological impairments in TBI, and that downregulating ZIPK expression protects against TBI-induced neuropathologies in multiple aspects.

### *Zipk* haploinsufficiency mitigates TBI-induced emotional and cognitive impairments

**Fig. 7:**
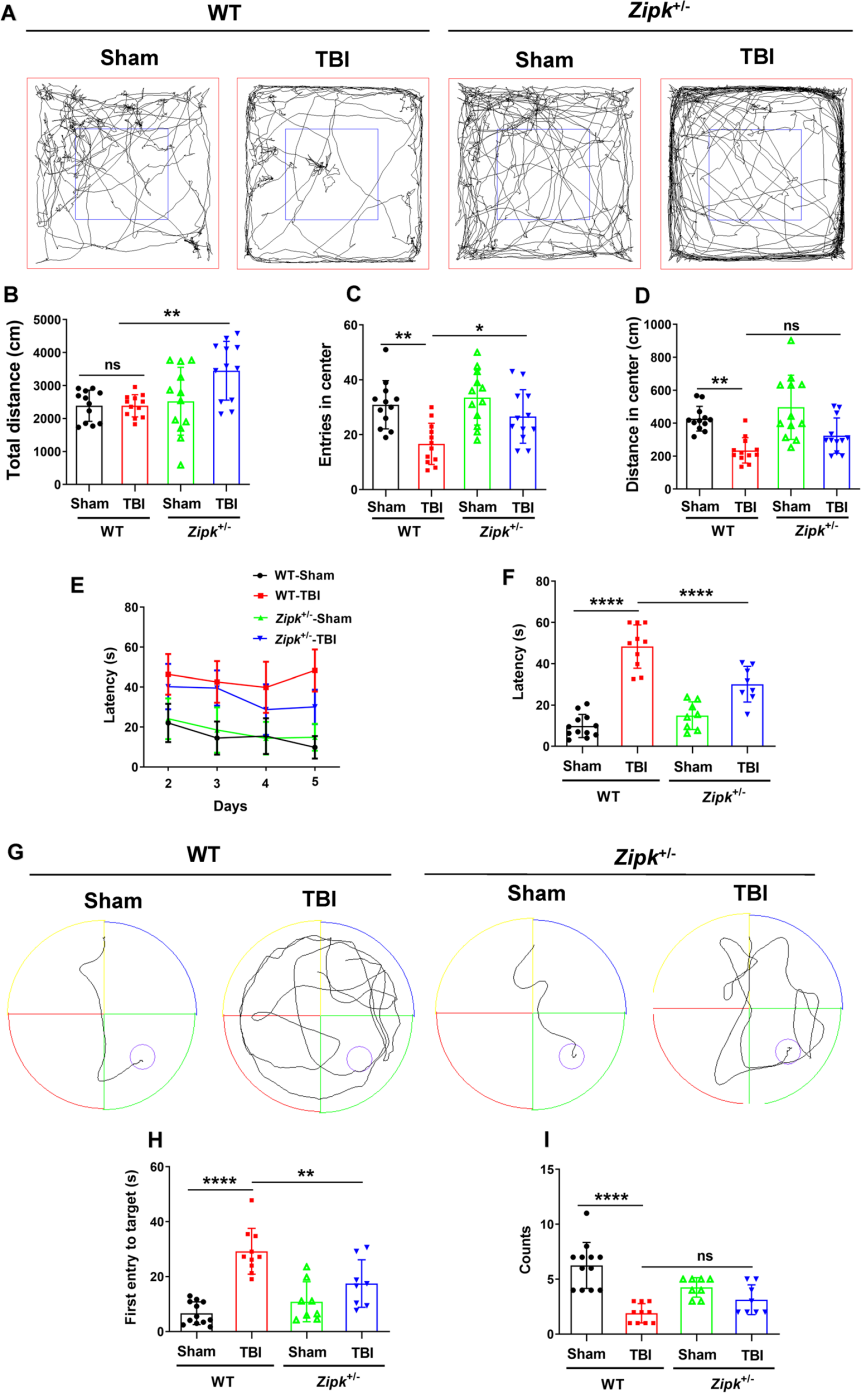
*Zipk* haploinsufficiency mitigates TBI-induced cognitive and emotional impairments. **A**–**D** WT and *Zipk*^+/−^ mice were subjected to the OFT at 4 days post TBI. Representative trajectories of mice in four groups (**A**), the total distance traveled (**B**), the number of entries to central zone, and the distance traveled in the central area (**C**, **D**) were measured and quantified. **E**–**I** Mice were tested in MWM to assess spatial learning and memory abilities 10 days after TBI. The learning curve in the training phase (**E**), escape latency at day 5 (**F**), and representative swimming trajectories (**G**) are presented. The time of the first entry to the platform (**H**) and the count of platform crossings (**I**) for all groups in the probe trial (day 6) were shown. *n* = 8–12 mice/group. *P < 0.05, **P < 0.01, ***P < 0.001, ****P < 0.0001, ns, not significant. One-way ANOVA was used for analysis.

TBI not only causes motor and sensory deficits but also exacerbates the emotional and cognitive functions chronically. As the mNSS analysis revealed the promising benefits of *Zipk* haploinsufficiency, we further studied the effect of ZIPK downregulation in vivo through OFT and MWM tests. We first measured the locomotor and anxiety-like behavior of mice in OFT. The distance traveled in the central area, and the entries to the central area were markedly lower in WT-TBI mice than in the WT-Sham mice (Fig. 7A–D), suggesting the induction of anxiety-like phenotype by CCI. Compared with WT-TBI group, the *Zipk*^+/−^-TBI group manifested increased entries to the central zone of the open field, while the distance in the center was comparable between the two groups (Fig. 7A–D), indicating more exploratory behavior in *Zipk*^+/−^-TBI mice. To determine whether *Zipk* haploinsufficiency can improve the cognitive dysfunction caused by TBI, MWM test was used to evaluate the spatial learning and memory abilities of all mice. Mice showed comparable swimming speeds in the visible platform test (Fig. S6). During the training phase of MWM (day 2 to day 5), the escape latency of WT-TBI group was longer than that of the sham mice, indicating that CCI caused notable cognitive dysfunction in mice (Fig. 7E–G). Nevertheless, there was a measurable difference in the escape latency on day 5 between *Zipk*^+/−^ and WT mice exposed to TBI (Fig. 7F). Besides, in the probe trial (day 6), there was a consistent trend toward improved cognitive function in *Zipk*^+/−^ compared with WT mice after TBI, as indicated by a significant decline in the time of first entry to the platform and a moderate increase in the target crossing counts (Fig. 7H, I). These data confirm that *Zipk* haploinsufficiency could moderately improve TBI-induced cognitive impairment under current experimental conditions. It should be mentioned that *Zipk*^+/−^-TBI mice had longer total ambulatory distance than WT-TBI mice in the OFT (Fig. 7B), while swimming speed in MWM was comparable between groups (Fig. S6). We reasoned that different measuring time point post TBI might account for the difference in motor activity between WT and *Zipk*^+/−^ mice in these two tests. Overall, by performing a set of behavioral tests, we verified that *Zipk* haploinsufficiency can mitigate TBI-induced cognitive and emotional impairments.

### ZIPK inhibition attenuates TBI-induced cell death by suppressing DEDD

**Fig. 8:**
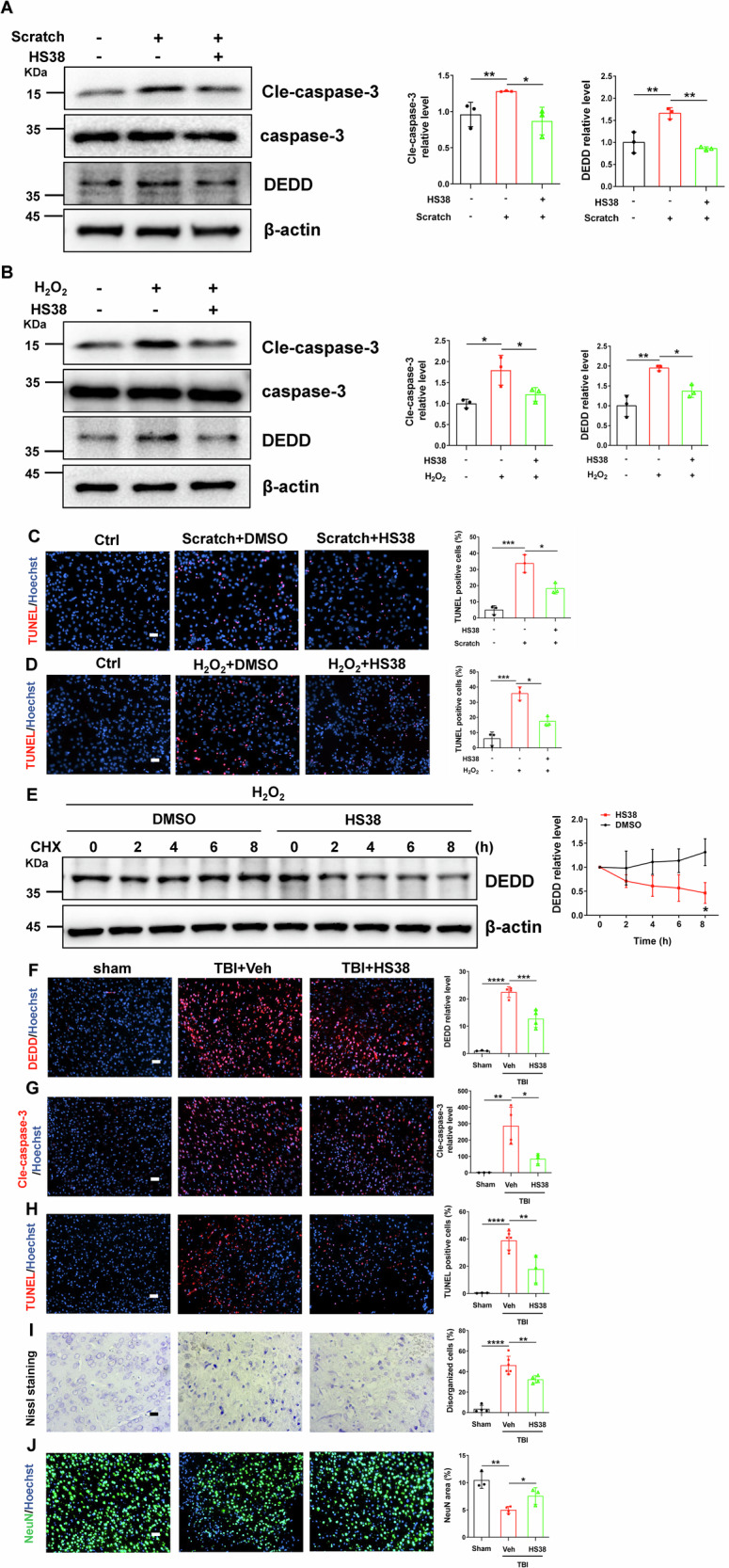
ZIPK inhibition attenuates TBI-induced cell death by suppressing DEDD. **A**, **B** Representative immunoblots of DEDD and cle-caspase-3 in WT primary neurons treated with or without HS38 2 h before scratch injury (**A**) or H_2_O_2_ (**B**) exposure. **C**, **D** TUNEL assay showing cell death in WT primary neurons treated with or without HS38 2 h before scratch (**C**) or H_2_O_2_ (**D**) exposure. The scale bar is 50 μm. **E** Primary neurons were pretreated with or without HS38 for 2 h, and exposed to 100 μM H_2_O_2_ for 24 h. Then CHX was added for indicated time points. A representative immunoblot of DEDD was shown. **F**, **G** Immunostaining of DEDD (**F**) and cle-caspase-3 (**G**) in peri-contusional brain areas of mice with or without HS38 pretreatment at 2 days post TBI. The scale bar is 50 μm. **H**–**J** TUNEL assay (**H**) (scale bar, 50 μm), Nissl staining (**I**) (scale bar, 20 μm) and NeuN staining (**J**) (scale bar, 50 μm) were performed to show the neuronal loss in peri-contusional brain areas of mice with or without HS38 pretreatment at 2 days post TBI. *n* = 3–6 mice/group. *P < 0.05, **P < 0.01, ***P < 0.001, ****P < 0.0001. One-way ANOVA was used for analysis in (**A**–**D**, **F**–**J**), and unpaired two-tailed Student’s *t* test was performed in (**E**).

The encouraging data from *Zipk*^+/−^ mice support ZIPK as an interventional target for TBI-induced neurological damage. To illustrate the potential of targeting ZIPK in the treatment of TBI-related neuropathologies, we utilized a well-established ZIPK inhibitor HS38 to suppress the kinase activity of ZIPK, and evaluated the effect of ZIPK inhibition on cellular and mouse TBI models [13, 44]. First, primary neurons were pretreated with or without HS38 for 2 h before exposure to scratch or H_2_O_2_. The results showed that the increase of DEDD and cle-caspase-3 induced by scratch or H_2_O_2_ was effectively inhibited after HS38 treatment (Fig. 8A, B). Moreover, TUNEL-positive signals in primary neurons were substantially decreased after the addition of HS38 (Fig. 8C, D). To explore whether the protective effect of HS38 is also mediated by DEDD regulation, we quantified the protein stability of endogenous DEDD after HS38 addition. We found that endogenous DEDD level remained stable within 8 h in the vehicle-treated cells, but the DEDD content in HS38-treated cells reduced to about 50% at 8 h (Fig. 8E). These results support our findings that ZIPK modulates the expression of DEDD by affecting its protein stability in a kinase activity-dependent manner.

Furthermore, we wanted to explore whether HS38 has similar protective effects in in vivo TBI model. We injected HS38 (10 mg/kg body weight) intraperitoneally half an hour before TBI, and then sacrificed all mice 2 days after TBI for pathological examination. Immunostaining analyses demonstrated that pretreatment with HS38 not only suppressed TBI-induced DEDD accumulation and caspase-3 activation in the brain (Fig. 8F, G) but also limited neuronal cell death and structural damage, as shown by TUNEL assay, Nissl staining and NeuN staining (Fig. 8H–J). These results are in accordance with what we have observed in the *Zipk*^+/−^ mice, and provide solid evidence for the future evaluation of ZIPK as a novel therapeutic target for TBI-related neuropathologies.

## Discussion

As a member of the DAPK family, ZIPK was initially suggested to mediate apoptosis, acting as a tumor suppressing-kinase to prevent malignant phenotypes in cancer [45, 46]. Recent studies uncovered a novel role for ZIPK in regulating ischemic stroke by affecting the normal functioning of endothelium in the brain, as ZIPK is abundantly expressed in endothelial cells [9, 47]. However, little is known about the expression and biological relevance of neuronal ZIPK. Here, we established that the expression of ZIPK is strongly elevated in neurons following TBI, and that ZIPK is closely involved in neuronal apoptosis through modulating the DEDD/caspase-3 pathway. Specifically, we propose that the deregulated DEDD function after TBI is likely due to ZIPK-induced DEDD phosphorylation and the subsequent stabilization.

Brain trauma triggers the dysregulation of diverse protein kinases in cells, resulting in the aberrant activity of signaling pathways responsible for cell survival, repair, and neural plasticity [48]. For example, TBI is known to activate stress-related protein kinases such as the mitogen-activated protein kinase family, thereby facilitating widespread cerebral damage through influencing neuronal or glial cell functions [49, 50]. We and others previously found that the expression of DAPK1, another member of DAPK family, is increased in a plethora of neurological diseases, including AD, ischemic stroke, and TBI [21, 51]. In particular, the upregulation of DAPK1 after TBI enhances the accumulation of *cis* P-tau, the main culprit of axonal injury in neurodegenerative diseases [21]. Nevertheless, whether and how DAPK family members contribute to neuronal loss in TBI have not been elucidated, although these proteins are highly relevant to cell apoptosis. Herein, we constructed in vivo and in vitro TBI models, and showed that ZIPK protein expression but not its mRNA level is rapidly upregulated in neurons after TBI (Fig. 1). Although Zhang et al. previously reported that ZIPK is predominately expressed in brain endothelial cells but not neurons [9], we were able to detect neuronal ZIPK expression by both immunoblotting and immunofluorescence, especially in peri-injury sites after TBI (Fig. 1E). We speculate that cellular stress caused by mechanical injury or oxidative damage could boost ZIPK expression in neurons, similar to what occurs during chronological aging and ionizing radiation treatment in human brain microvascular endothelial cells [11]. However, in contrast with the transcriptional upregulation of ZIPK in endothelial cells exposed to ionizing radiation, we did not observe any change in the mRNA level of *ZIPK* in neurons underwent TBI (Fig. 1J–N), indicating that neuronal ZIPK is dysregulated post-transcriptionally. The exact mechanism underlying ZIPK upregulation in TBI remains to be elucidated, but this finding agrees with a previous study showing that ZIPK expression is pronouncedly elevated in the frontal cortices of AD patients [10].

Neuronal cell death is a prominent feature of TBI. Multiple cell death pathways are dysregulated after brain trauma, making it challenging to address neuronal loss and subsequent sequelae in the clinical management of TBI [52]. However, several clinical research has developed apoptotic markers such as caspase-3 to indicate the short- or long-term outcomes of brain injury in patients [4]. We were able to show the activation of caspase-3 in peri-contusional regions of TBI mice at both 2 days (Fig. 8G) and 16 days (Fig. 6D) after injury, as well as in cellular TBI models (Fig. 3). The hyperactivation of caspase-3 is a direct initiator of neuronal apoptosis, as evidenced by the marked increase in TUNEL-positive signals and the loss of NeuN staining in the cortex of TBI mice (Fig. 2A, C). Excessive neuronal apoptosis further leads to structural damage shown by the formation of disorganized Nissl bodies (Fig. 2B) and the disappearance of MAP2 and NFL (Fig. 6E, F), indicating the degeneration of neurons after TBI. As a pro-apoptotic protein, ZIPK induces cell death by involving caspase-dependent apoptotic pathway or caspase-independent autophagic pathway [8, 36]. The inhibition of neuronal cell death by *Zipk* haploinsufficiency or ZIPK inhibitor treatment in our TBI models was preceded by a dramatic reduction in the expression of cle-caspase-3 (Figs. 6D and 8G), emphasizing the importance of caspase-3-dependent apoptotic pathway in mediating ZIPK upregulation-induced neuronal loss in TBI. Moreover, the preservation of neuronal viability by *Zipk* haploinsufficiency was accompanied by a notable recovery in the expression of axonal and synaptic markers in vivo (Fig. 6E, F), which could explain the attenuation of neurobehavioral deficits in *Zipk*^+/−^ mice underwent TBI. Interestingly, the upregulation of ZIPK in the cortex of AD patients is associated with the dysregulation of apoptosis-related proteins such as the Bcl-2 protein family members and prostate apoptosis response-4 (Par-4) [10, 53], all of which are important factors in the neuronal apoptosis cascade. These data together strengthen our key findings that ZIPK is a principal contributor to promoting neuronal cell death in neurological disorders including TBI. However, given the complex pathophysiology of TBI, ZIPK might also play a part in other types of cell death such as autophagic cell death, ferroptosis and necrosis, which warrants further investigation both in vivo and in vitro.

Kinase activity plays an indispensable role in modulating the pathophysiological functions of ZIPK. For example, cellular stress such as arsenic trioxide or interferon-gamma treatment can cause apoptosis through ZIPK-mediated substrate phosphorylation [31, 54, 55]. The phosphorylation of Par-4 by ZIPK at the T155 residue induces apoptosis in rat fibroblasts, which can be blocked by T155A mutation or inactivation of ZIPK [54]. In addition, the regulatory effect of ZIPK on cellular autophagy also relies on ZIPK-mediated direct phosphorylation of Beclin1 or UNC-51-like kinase 1 [36, 56]. Thus, ZIPK-induced protein phosphorylation is proposed to be a leading mechanism in cell death pathways. To identify how ZIPK modulates the function of caspase-3 in neurons, we focused on DEDD, a scaffold protein that directs active caspase-3 to its substrates during apoptosis [33]. We found that DEDD expression in neurons is post-transcriptionally controlled by ZIPK in cellular and animal TBI models (Figs. 4A–F and S3). In addition, DEDD is a direct substrate of ZIPK and was found to be necessary for ZIPK-induced neuronal loss in TBI models, as DEDD knockdown abolished the influence of ZIPK on caspase-3 activation and apoptosis (Fig. 3E, F). ZIPK-induced DEDD S9 phosphorylation increased DEDD protein stability, as revealed by the decreased DEDD degradation in the presence of ZIPK (Fig. 4G, H) and the accelerated DEDD degradation caused by DEDD-S9A mutation (Fig. 5F) or HS38 treatment (Fig. 8E). Moreover, the nonphosphorylatable DEDD-S9A mutant efficiently alleviated neuronal cell death irrespective of the presence of ZIPK (Fig. 5G–I), indicating that DEDD phosphorylation at S9 may, in addition to stabilizing DEDD protein, also downregulate its pro-apoptotic activity. These results thus support that DEDD phosphorylation at S9 serves as an upstream regulator of caspase-3-dependent apoptosis in TBI. The detailed mechanisms by which DEDD phosphorylation at S9 affects its protein stability and activity have yet to be explored. Protein phosphorylation can either activate or deactivate the degron signal motifs, thereby affecting protein stability and degradation [57]. It is possible that the phosphorylation of DEDD at S9 stabilizes DEDD by producing an inactivated degron, masking the original degron in DEDD, or altering the subcellular localization of DEDD. Besides, the S9 residue is close to the nuclear localization signals and the death effector domain of DEDD, two essential components responsible for its regulation on caspase-3 activation. We speculate that S9 phosphorylation may negatively impact the interplay between DEDD and caspase-3, therefore affecting neuronal apoptosis. Although we proved that TBI-induced DEDD accumulation could be reversed by *Zipk* haploinsufficiency or HS38 treatment in vivo, we could not detect the phosphorylation of DEDD at S9 in mouse brain due to the lack of phospho-specific antibody. Future studies are needed to validate DEDD phosphorylation in TBI mouse models and potentially in TBI patient samples.

The pathological significance of ZIPK in the brain is less studied than that of DAPK1, likely due to differences in the expression patterns. DAPK1 is highly expressed at neuronal synapses, while ZIPK is mainly detected in brain endothelial cells according to previous research [9, 58]. ZIPK modulates endothelial cell contraction by phosphorylating the myosin light chain, thereby affecting the endothelium permeability important for maintaining BBB integrity [47]. The Evans blue assay demonstrated that *Zipk*^+/−^ mice had less BBB leakage compared with WT mice after brain injury (Fig. 6I), which is consistent with the protection of endothelial-induced deletion of ZIPK against ischemia–reperfusion damage in mice [9]. Since ZIPK was globally downregulated in *Zipk*^+/−^ mice, we could not exclude the possibility that the preservation of BBB integrity was caused by reduced expression of ZIPK in the endothelium. Apart from characterizing neurite markers directly linked to neuronal survival, we have also analyzed the activation of glial cells in the mouse models, although there was no evident colocalization between ZIPK and GFAP or Iba1 (Fig. 1E). Activation of astrocytes and microglia is an acute and widespread response toward TBI-induced brain damage in the injury area [59]. The breakdown of BBB also exacerbates glial activation by recruiting neutrophils and other immune factors to the injury site, all of which contribute to the focal inflammatory response in TBI [60, 61]. We observed a considerable decrease in GFAP levels (Fig. 6G), and a descending trend in Iba1 signals in *Zipk*^+/−^ mice after CCI injury. We hypothesize that the attenuation of glial activation after ZIPK knockdown might be attributed to indirect mechanisms such as reduced neuronal damage or BBB breakdown in *Zipk*^+/−^ mice.

TBI is known to cause short-term and long-lasting behavioral impairments including motor and sensory deficits and neurocognitive sequelae [62, 63]. Anxiety-like behavior and memory dysfunction are two common phenotypes in rodent TBI models [64]. *Zipk* haploinsufficiency ameliorated TBI-induced anxiety-like phenotype by increasing the exploratory behavior in the open field test (Fig. 7A–D). There was also a moderate but significant improvement in spatial learning and memory behavior in *Zipk*^+/−^-TBI mice (Fig. 7E–I). The cognitive recovery is accompanied by a reduction in neuronal loss and the restoration of dendritic and axonal integrity. The beneficial effect of *Zipk* haploinsufficiency on CCI-induced behavioral impairments is similar to the effect of DAPK1 knockout on long-term cognitive dysfunction in a weight-drop TBI model [21]. We performed analyses for up to 16 days after TBI in the present study, whereas we assessed behavioral phenotypes at 3.5 and 5 months after TBI in the previous study [21]. In the HS38 treatment experiment, we pretreated the mice with ZIPK inhibitor once and determined the efficacy in the acute phase after injury (2 days). HS38 is able to suppress DAPK1 due to the highly homologous kinase domains in DAPK family proteins. Besides, DAPK1 is upregulated after TBI. Although the data from *Zipk*^+/−^ mice and ZIPK knockdown cells support that partial deletion of ZIPK effectively ameliorated neuronal loss and cognitive impairment, we cannot exclude the potential contribution of DAPK1 inhibition by HS38 in the inhibitor treatment experiments. Further studies are needed to dissect the distinct role of DAPK1 and ZIPK in the pathogenesis of TBI. The long-term effects of ZIPK downregulation or kinase inhibition on TBI-induced cognitive dysfunction warrants further analysis. Notably, *Zipk*^+/−^ mice performed identically to WT mice in all the behavioral tests, indicating that *Zipk* haploinsufficiency is unlikely to negatively affect the overall neurological function, which aligns well with the comparable brain morphology and body size between two genotypes. Thus, *Zipk* haploinsufficiency or conditional knockout could be a viable approach for investigating the pathophysiological role of ZIPK in neurological disorders [30]. Besides, considering the vital function of ZIPK in non-neuronal cells, strategies for the targeted delivery of highly selective ZIPK inhibitors are needed for TBI treatment in the future.

**Fig. 9:**
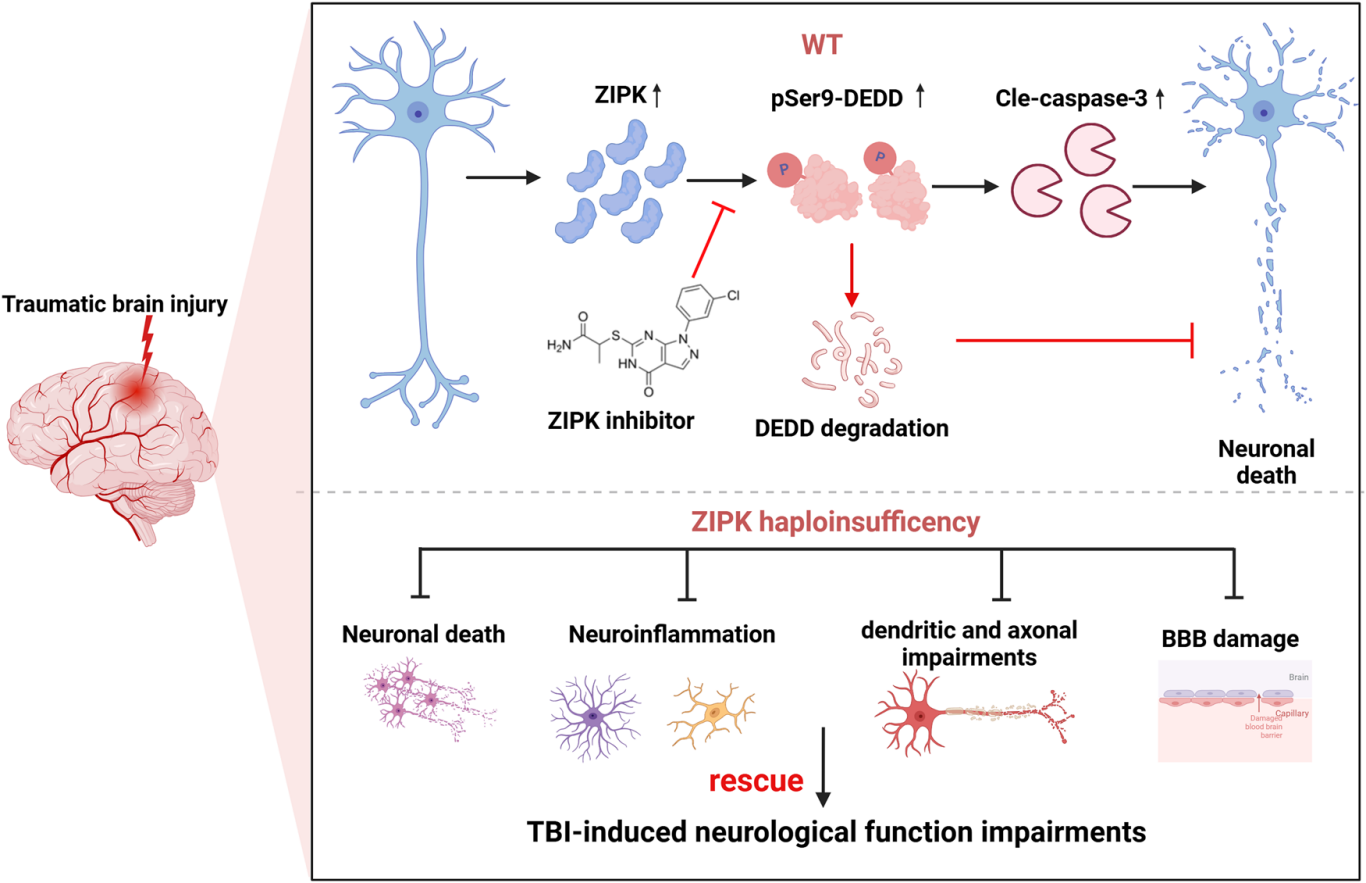
Schematic representation of the pathological role of ZIPK in TBI-induced neuronal cell death. The upregulation of ZIPK expression caused by brain trauma promotes cell death by triggering neuronal damage through phosphorylating DEDD at S9, leading to the accumulation of DEDD and the activation of caspase-3-mediated apoptosis, which ultimately impairs brain function and results in cognitive deterioration. *Zipk* haploinsufficiency or inhibition of ZIPK activity by HS38 effectively promotes neuronal survival by inhibiting DEDD phosphorylation and caspase-3 activity after TBI, thereby rescuing TBI-induced neuronal damage and behavioral impairments. Figure was created using BioRender.

Collectively, our results suggest a fundamental role for neuronal ZIPK in regulating TBI-related cell apoptosis and neuropathology (Fig. 9). In brief, the upregulation of ZIPK after TBI leads to neuronal cell death by modulating the DEDD/caspase-3 cascade. ZIPK phosphorylates and stabilizes DEDD, thereby facilitating the activation of caspase-3-mediated apoptotic pathways in neurons, further leading to synaptic degeneration, BBB breakdown, and gliosis. Downregulating ZIPK expression or pharmacological inhibition confers protections against neuronal loss by normalizing the DEDD/caspase-3 pathway, and improves the neurobehavioral outcomes in TBI models. These data provide a basis for further investigations of ZIPK as a novel therapeutic target for the treatment of brain trauma and neurological diseases.

## Supplementary information

Supplementary information

Full and uncropped Western blots

## Data Availability

Data supporting the conclusions in the paper are presented in the main text and the Supplementary Materials. Additional data are available from the corresponding authors on request.

## References

[1] Maas AIR, Menon DK, Manley GT, Abrams M, Akerlund C, Andelic N, et al. Traumatic brain injury: progress and challenges in prevention, clinical care, and research. Lancet Neurol. 2022;21:1004–60. 36183712 10.1016/S1474-4422(22)00309-X PMC10427240

[2] Brett BL, Gardner RC, Godbout J, Dams-O’Connor K, Keene CD. Traumatic brain injury and risk of neurodegenerative disorder. Biol Psychiatry. 2022;91:498–507. 34364650 10.1016/j.biopsych.2021.05.025 PMC8636548

[3] Jassam YN, Izzy S, Whalen M, McGavern DB, El Khoury J. Neuroimmunology of traumatic brain injury: time for a paradigm shift. Neuron. 2017;95:1246–65. 28910616 10.1016/j.neuron.2017.07.010 PMC5678753

[4] Akamatsu Y, Hanafy KA. Cell death and recovery in traumatic brain injury. Neurotherapeutics. 2020;17:446–56. 32056100 10.1007/s13311-020-00840-7 PMC7283441

[5] Bae YH, Joo H, Bae J, Hyeon SJ, Her S, Ko E, et al. Brain injury induces HIF-1alpha-dependent transcriptional activation of LRRK2 that exacerbates brain damage. Cell Death Dis. 2018;9:1125. 30420654 10.1038/s41419-018-1180-y PMC6232134

[6] Wehn AC, Khalin I, Duering M, Hellal F, Culmsee C, Vandenabeele P, et al. RIPK1 or RIPK3 deletion prevents progressive neuronal cell death and improves memory function after traumatic brain injury. Acta Neuropathol Commun. 2021;9:138. 34404478 10.1186/s40478-021-01236-0 PMC8369637

[7] Bao Z, Liu Y, Chen B, Miao Z, Tu Y, Li C, et al. Prokineticin-2 prevents neuronal cell deaths in a model of traumatic brain injury. Nat Commun. 2021;12:4220. 34244497 10.1038/s41467-021-24469-y PMC8270965

[8] Usui T, Okada M, Yamawaki H. Zipper interacting protein kinase (ZIPK): function and signaling. Apoptosis. 2014;19:387–91. 24193917 10.1007/s10495-013-0934-3

[9] Zhang Y, Zhang C, Zhang H, Zeng W, Li S, Chen C, et al. ZIPK mediates endothelial cell contraction through myosin light chain phosphorylation and is required for ischemic-reperfusion injury. FASEB J. 2019;33:9062–74. 31180722 10.1096/fj.201802052RRR PMC6662964

[10] Engidawork E, Gulesserian T, Seidl R, Cairns N, Lubec G. Expression of apoptosis related proteins in brains of patients with Alzheimer’s disease. Neurosci Lett. 2001;303:79–82. 11311497 10.1016/s0304-3940(01)01618-4

[11] Park JE, Park JW, Sim MK, Kim SR, Kim KS. Inhibition of DAPK3 suppresses radiation-induced cellular senescence by activation of a PGC1alpha-dependent metabolism pathway in brain endothelial cells. J Gerontol A Biol Sci Med Sci. 2024;79:glae088. 10.1093/gerona/glae088 38563090

[12] Wilson L, Stewart W, Dams-O’Connor K, Diaz-Arrastia R, Horton L, Menon DK, et al. The chronic and evolving neurological consequences of traumatic brain injury. Lancet Neurol. 2017;16:813–25. 28920887 10.1016/S1474-4422(17)30279-X PMC9336016

[13] MacDonald JA, Sutherland C, Carlson DA, Bhaidani S, Al-Ghabkari A, Sward K, et al. A small molecule pyrazolo[3,4-d]pyrimidinone inhibitor of zipper-interacting protein kinase suppresses calcium sensitization of vascular smooth muscle. Mol Pharm. 2016;89:105–17. 10.1124/mol.115.100529 26464323

[14] Wu Y, Wu H, Zeng J, Pluimer B, Dong S, Xie X, et al. Mild traumatic brain injury induces microvascular injury and accelerates Alzheimer-like pathogenesis in mice. Acta Neuropathol Commun. 2021;9:74. 33892818 10.1186/s40478-021-01178-7 PMC8063402

[15] Xue Y, Zhang Y, Wu Y, Zhao T. Activation of GPER-1 attenuates traumatic brain injury-induced neurological impairments in mice. Mol Neurobiol. 2024;61:5614–27. 10.1007/s12035-024-03919-w 38217667

[16] Zhang T, Xia Y, Hu L, Chen D, Gan CL, Wang L, et al. Death-associated protein kinase 1 mediates Abeta42 aggregation-induced neuronal apoptosis and tau dysregulation in Alzheimer’s disease. Int J Biol Sci. 2022;18:693–706. 35002518 10.7150/ijbs.66760 PMC8741852

[17] Chen D, Mei Y, Kim N, Lan G, Gan CL, Fan F, et al. Melatonin directly binds and inhibits death-associated protein kinase 1 function in Alzheimer’s disease. J Pineal Res. 2020;69:e12665. 32358852 10.1111/jpi.12665 PMC7890046

[18] Livak KJ, Schmittgen TD. Analysis of relative gene expression data using real-time quantitative PCR and the 2(-delta delta C(T)) method. Methods. 2001;25:402–8. 11846609 10.1006/meth.2001.1262

[19] You MH, Kim BM, Chen CH, Begley MJ, Cantley LC, Lee TH. Death-associated protein kinase 1 phosphorylates NDRG2 and induces neuronal cell death. Cell Death Differ. 2017;24:238–50. 28141794 10.1038/cdd.2016.114 PMC5299707

[20] Zhang M, Shui X, Zheng X, Lee JE, Mei Y, Li R, et al. Death-associated protein kinase 1 phosphorylates MDM2 and inhibits its protein stability and function. Arch Pharm Res. 2023;46:882–96. 37804415 10.1007/s12272-023-01469-8

[21] Kim N, Wang B, Koikawa K, Nezu Y, Qiu C, Lee TH, et al. Inhibition of death-associated protein kinase 1 attenuates cis P-tau and neurodegeneration in traumatic brain injury. Prog Neurobiol. 2021;203:102072. 33979671 10.1016/j.pneurobio.2021.102072 PMC8217320

[22] Gan CL, Zou Y, Xia Y, Zhang T, Chen D, Lan G, et al. Inhibition of death-associated protein kinase 1 protects against epileptic seizures in mice. Int J Biol Sci. 2021;17:2356–66. 34239362 10.7150/ijbs.59922 PMC8241737

[23] Mi L, Min X, Shi M, Liu L, Zhang Y, Zhu Y, et al. Neutrophil extracellular traps aggravate neuronal endoplasmic reticulum stress and apoptosis via TLR9 after traumatic brain injury. Cell Death Dis. 2023;14:374. 37365190 10.1038/s41419-023-05898-7 PMC10293297

[24] Zhang JM, Jing Y, Wang K, Jiao JT, Xu JY, Shi J, et al. Inhibition of heat shock protein 90 attenuates the damage of blood-brain barrier integrity in traumatic brain injury mouse model. Oxid Med Cell Longev. 2022;2022:5585384. 35450406 10.1155/2022/5585384 PMC9018170

[25] Zhou H, Yi Z, Le D, Mao G, Zhang H. Intravenous administration of human chorionic membrane mesenchymal stem cells promotes functional recovery in a rat traumatic brain injury model. Neuroreport. 2024;35:81–9. 38109419 10.1097/WNR.0000000000001981

[26] Flierl MA, Stahel PF, Beauchamp KM, Morgan SJ, Smith WR, Shohami E. Mouse closed head injury model induced by a weight-drop device. Nat Protoc. 2009;4:1328–37. 19713954 10.1038/nprot.2009.148

[27] Wang R, Chu C, Wei Z, Chen L, Xu J, Liang Y, et al. Traumatic brain injury does not disrupt costimulatory blockade-induced immunological tolerance to glial-restricted progenitor allografts. J Neuroinflammation. 2021;18:104. 33931070 10.1186/s12974-021-02152-9 PMC8088005

[28] Zhang T, Tian Y, Zheng X, Li R, Hu L, Shui X, et al. Activation of transient receptor potential vanilloid 1 ameliorates tau accumulation-induced synaptic damage and cognitive dysfunction via autophagy enhancement. CNS Neurosci Ther. 2024;30:e14432. 37641913 10.1111/cns.14432 PMC10916438

[29] Dong W, Gong F, Zhao Y, Bai H, Yang R. Ferroptosis and mitochondrial dysfunction in acute central nervous system injury. Front Cell Neurosci. 2023;17:1228968. 37622048 10.3389/fncel.2023.1228968 PMC10445767

[30] Kocher BA, White LS, Piwnica-Worms D. DAPK3 suppresses acini morphogenesis and is required for mouse development. Mol Cancer Res. 2015;13:358–67. 25304685 10.1158/1541-7786.MCR-14-0333 PMC4336824

[31] Kawai T, Akira S, Reed JC. ZIP kinase triggers apoptosis from nuclear PML oncogenic domains. Mol Cell Biol. 2003;23:6174–86. 12917339 10.1128/MCB.23.17.6174-6186.2003 PMC180930

[32] Unnisa A, Greig NH, Kamal MA. Inhibition of caspase 3 and caspase 9 mediated apoptosis: a multimodal therapeutic target in traumatic brain injury. Curr Neuropharmacol. 2023;21:1001–12. 35339178 10.2174/1570159X20666220327222921 PMC10227914

[33] Lee JC, Schickling O, Stegh AH, Oshima RG, Dinsdale D, Cohen GM, et al. DEDD regulates degradation of intermediate filaments during apoptosis. J Cell Biol. 2002;158:1051–66. 12235123 10.1083/jcb.200112124 PMC2173221

[34] Schutte B, Henfling M, Ramaekers FC. DEDD association with cytokeratin filaments correlates with sensitivity to apoptosis. Apoptosis. 2006;11:1561–72. 16820959 10.1007/s10495-006-9113-0

[35] Webster JA, Gibbs JR, Clarke J, Ray M, Zhang W, Holmans P, et al. Genetic control of human brain transcript expression in Alzheimer disease. Am J Hum Genet. 2009;84:445–58. 19361613 10.1016/j.ajhg.2009.03.011 PMC2667989

[36] Li GM, Li L, Li MQ, Chen X, Su Q, Deng ZJ, et al. DAPK3 inhibits gastric cancer progression via activation of ULK1-dependent autophagy. Cell Death Differ. 2021;28:952–67. 33037394 10.1038/s41418-020-00627-5 PMC7937684

[37] Halbgebauer R, Halbgebauer S, Oeckl P, Steinacker P, Weihe E, Schafer MK, et al. Neurochemical monitoring of traumatic brain injury by the combined analysis of plasma beta-synuclein, NfL, and GFAP in polytraumatized patients. Int J Mol Sci. 2022;23:9639. 10.3390/ijms23179639 PMC9456193 36077033

[38] Mirro J, Dow LW, Kalwinsky DK, Dahl GV, Weck P, Whisnant J, et al. Phase I-II study of continuous-infusion high-dose human lymphoblastoid interferon and the in vitro sensitivity of leukemic progenitors in nonlymphocytic leukemia. Cancer Treat Rep. 1986;70:363–7. 3456833

[39] Ozen I, Arkan S, Clausen F, Ruscher K, Marklund N. Diffuse traumatic injury in the mouse disrupts axon-myelin integrity in the cerebellum. J Neurotrauma. 2022;39:411–22. 35018831 10.1089/neu.2021.0321

[40] Qin D, Wang J, Le A, Wang TJ, Chen X, Wang J. Traumatic brain injury: ultrastructural features in neuronal ferroptosis, glial cell activation and polarization, and blood-brain barrier breakdown. Cells. 2021;10:1009. 10.3390/cells10051009 PMC8146242 33923370

[41] Zhao Q, Li H, Li H, Xie F, Zhang J. Research progress of neuroinflammation-related cells in traumatic brain injury: a review. Medicine. 2023;102:e34009. 37352020 10.1097/MD.0000000000034009 PMC10289497

[42] Zeng W, Sun Z, Ma T, Song X, Li S, Zhang Q, et al. Elevated ZIPK is required for TNF-alpha-induced cell adhesion molecule expression and leucocyte adhesion in endothelial cells. Acta Biochim Biophys Sin. 2021;53:567–74. 33710297 10.1093/abbs/gmab019

[43] Usami S, Hozawa J, Tazawa M, Igarashi M, Thompson GC, Wu JY, et al. Immunocytochemical study of the GABA system in chicken vestibular endorgans and the vestibular ganglion. Brain Res. 1989;503:214–8. 2605515 10.1016/0006-8993(89)91666-1

[44] Carlson DA, Singer MR, Sutherland C, Redondo C, Alexander LT, Hughes PF, et al. Targeting pim kinases and DAPK3 to control hypertension. Cell Chem Biol. 2018;25:1195–207.e32. 30033129 10.1016/j.chembiol.2018.06.006 PMC6863095

[45] Bi J, Lau SH, Hu L, Rao HL, Liu HB, Zhan WH, et al. Downregulation of ZIP kinase is associated with tumor invasion, metastasis and poor prognosis in gastric cancer. Int J Cancer. 2009;124:1587–93. 19117059 10.1002/ijc.24164

[46] Brognard J, Zhang YW, Puto LA, Hunter T. Cancer-associated loss-of-function mutations implicate DAPK3 as a tumor-suppressing kinase. Cancer Res. 2011;71:3152–61. 21487036 10.1158/0008-5472.CAN-10-3543 PMC3078168

[47] Gu Z, Li S, Liu J, Zhang X, Pang C, Ding L, et al. Protection of blood-brain barrier by endothelial DAPK1 deletion after stroke. Biochem Biophys Res Commun. 2024;724:150216. 38851140 10.1016/j.bbrc.2024.150216

[48] Sharma HS, Sahib S, Tian ZR, Muresanu DF, Nozari A, Castellani RJ, et al. Protein kinase inhibitors in traumatic brain injury and repair: new roles of nanomedicine. Prog Brain Res. 2020;258:233–83. 33223036 10.1016/bs.pbr.2020.09.009

[49] Mori T, Wang X, Jung JC, Sumii T, Singhal AB, Fini ME, et al. Mitogen-activated protein kinase inhibition in traumatic brain injury: in vitro and in vivo effects. J Cereb Blood Flow Metab. 2002;22:444–52. 11919515 10.1097/00004647-200204000-00008

[50] Rehman SU, Ahmad A, Yoon GH, Khan M, Abid MN, Kim MO. Inhibition of c-Jun N-terminal kinase protects against brain damage and improves learning and memory after traumatic brain injury in adult mice. Cereb Cortex. 2018;28:2854–72. 29088310 10.1093/cercor/bhx164

[51] Zhang T, Kim BM, Lee TH. Death-associated protein kinase 1 as a therapeutic target for Alzheimer’s disease. Transl Neurodegener. 2024;13:4. 38195518 10.1186/s40035-023-00395-5 PMC10775678

[52] Stoica BA, Faden AI. Cell death mechanisms and modulation in traumatic brain injury. Neurotherapeutics. 2010;7:3–12. 20129492 10.1016/j.nurt.2009.10.023 PMC2841970

[53] Wang G, Dinkins M, He Q, Zhu G, Poirier C, Campbell A, et al. Astrocytes secrete exosomes enriched with proapoptotic ceramide and prostate apoptosis response 4 (PAR-4): potential mechanism of apoptosis induction in Alzheimer disease (AD). J Biol Chem. 2012;287:21384–95. 22532571 10.1074/jbc.M112.340513 PMC3375560

[54] Boosen M, Vetterkind S, Kubicek J, Scheidtmann KH, Illenberger S, Preuss U. Par-4 is an essential downstream target of DAP-like kinase (Dlk) in Dlk/Par-4-mediated apoptosis. Mol Biol Cell. 2009;20:4010–20. 19625447 10.1091/mbc.E09-02-0173 PMC2743620

[55] Sato N, Kawai T, Sugiyama K, Muromoto R, Imoto S, Sekine Y, et al. Physical and functional interactions between STAT3 and ZIP kinase. Int Immunol. 2005;17:1543–52. 16219639 10.1093/intimm/dxh331

[56] Fujiwara N, Usui T, Ohama T, Sato K. Regulation of beclin 1 protein phosphorylation and autophagy by protein phosphatase 2A (PP2A) and death-associated protein kinase 3 (DAPK3). J Biol Chem. 2016;291:10858–66. 26994142 10.1074/jbc.M115.704908 PMC4865930

[57] Lee JM, Hammaren HM, Savitski MM, Baek SH. Control of protein stability by post-translational modifications. Nat Commun. 2023;14:201. 36639369 10.1038/s41467-023-35795-8 PMC9839724

[58] Goodell DJ, Zaegel V, Coultrap SJ, Hell JW, Bayer KU. DAPK1 Mediates LTD by Making CaMKII/GluN2B Binding LTP Specific. Cell Rep. 2017;19:2231–43. 28614711 10.1016/j.celrep.2017.05.068 PMC5549467

[59] Shi K, Zhang J, Dong JF, Shi FD. Dissemination of brain inflammation in traumatic brain injury. Cell Mol Immunol. 2019;16:523–30. 30846842 10.1038/s41423-019-0213-5 PMC6804599

[60] Jin J, Wang F, Tian J, Zhao X, Dong J, Wang N, et al. Neutrophil extracellular traps contribute to coagulopathy after traumatic brain injury. JCI Insight. 2023;8:e141110. 10.1172/jci.insight.141110 PMC10070118 36802340

[61] Shi G, Liu L, Cao Y, Ma G, Zhu Y, Xu J, et al. Inhibition of neutrophil extracellular trap formation ameliorates neuroinflammation and neuronal apoptosis via STING-dependent IRE1alpha/ASK1/JNK signaling pathway in mice with traumatic brain injury. J Neuroinflammation. 2023;20:222. 37777772 10.1186/s12974-023-02903-w PMC10543875

[62] Li LM, Carson A, Dams-O’Connor K. Psychiatric sequelae of traumatic brain injury—future directions in research. Nat Rev Neurol. 2023;19:556–71. 37591931 10.1038/s41582-023-00853-8

[63] Zhou C, Sun P, Hamblin MH, Yin KJ. Genetic deletion of Kruppel-like factor 11 aggravates traumatic brain injury. J Neuroinflammation. 2022;19:281. 36403074 10.1186/s12974-022-02638-0 PMC9675068

[64] Li Z, Yu S, Li L, Zhou C, Wang L, Tang S, et al. TREM2 alleviates white matter injury after traumatic brain injury in mice might be mediated by regulation of DHCR24/LXR pathway in microglia. Clin Transl Med. 2024;14:e1665. 38649789 10.1002/ctm2.1665 PMC11035381

