# Supplementary material for: Zipper-interacting protein kinase mediates neuronal cell death and cognitive dysfunction in traumatic brain injury via regulating DEDD
Source: Cell Death Dis. 2025 Mar 4;16(1):151. doi: 10.1038/s41419-025-07474-7 (PMC11876612; doi:10.1038/s41419-025-07474-7)
Supplement: Supplementary file 1 — Supplementary information [file 41419_2025_7474_MOESM1_ESM.pdf]

Supplementary information

Zipper-interacting protein kinase mediates neuronal cell death and cognitive dysfunction in traumatic brain injury via regulating DEDD

Yingxue Mei[1], Fei She[1], Ling Zhang[1], Gamin Kim[2], Ruomeng Li[1], Xiuzhi Zheng[1], Zonghai Wang[1], Renxuan Chen[1], Long Wang[1], Dongmei Chen[1], Jungho Kim[2], Tao Zhang[1, *], Tae Ho Lee[1, **]

[1]Fujian Key Laboratory of Translational Research in Cancer and Neurodegenerative Diseases
Institute of Basic Medicine
School of Basic Medical Sciences
Fujian Medical University
Fuzhou 350122, China
[2]Laboratory of Molecular and Cellular Biology
Department of Life Science
Sogang University
Seoul 04107, Korea

Running title: ZIPK dysregulation facilitates neuronal loss in TBI

*Corresponding author: Tao Zhang, Fujian Medical University, 1 Xuefu North Road, Fuzhou, Fujian 350122, China; E-mail: taozh@fjmu.edu.cn; Tel.: +86-591-2286-2498; Fax: +86-591-2286-2320.
**Corresponding author: Tae Ho Lee, Fujian Medical University, 1 Xuefu North Road, Fuzhou, Fujian 350122, China; E-mail: tlee0813@fjmu.edu.cn; Tel.: +86-591-2286-2498; Fax: +86-591-2286-2320.

Table S1. Information of primary antibodies used in the present study.

Antibody	Dilution	Source	Identifier
Mouse anti-β-actin	1: 40000	Sigma-Aldrich	A5441
Rabbit anti-ZIPK	1: 2000	Abcam	ab210528
Mouse anti-NeuN	1: 1000	Millipore	MAB377
Mouse anti-GFAP	1: 500	Santa Cruz Biotechnology	sc-33673
Mouse anti-Iba1	1: 1000	Abcam	ab283319
Mouse anti-DEDD	1: 200	Santa Cruz Biotechnology	sc-271192
Rabbit anti-Cleaved-caspase-3 (Asp175)	1: 500	Cell Signaling Technology	9661
Rabbit anti-Caspase-3	1:1000	Abcam	ab13847
Rabbit anti-Caspase-3	1:1000	Cell Signaling Technology	9662
Rabbit-anti-Phospho - (Ser/Thr) Phe	1: 3000	Abcam	ab300625
Rabbit anti MAP2	1: 500	Cell Signaling Technology	4542
Rabbit anti-Neurofilament-L	1: 100	Cell Signaling Technology	2837
Mouse anti-Flag-tag	1: 3000	Cell Signaling Technology	8146
Mouse anti-HA-tag	1: 1000	Cell Signaling Technology	2367

Table S2. Sequence information of siRNA.

si-RNA	sequence
Human si-ZIPK	CAGAGAUUGUGAACUAUGAdTdT
	UCAUAGUUCACAAUCUCUGdTdT
Human si-DEDD	CCAUCAAGCUGCUGGUAAATT
	UUUACCAGCAGCUUGAUGGTT
Negative control (NC)	UUCUCCGAACGUGUCACGU TT
	ACGUGACACGUUCGGAGAA TT

Table S3. Sequence information of genotyping and qRT-PCR.

Primers-genotyping	5'-3'
Genotyping *Zipk*-1-F	TTTGAGCCGAGTCCTTGAGCT
Genotyping *Zipk*-1-R	ATAGGGACCTCAAGTGAGAGTCTGC
Genotyping *Zipk*-2-F	GGAACATCATAGGCCAGAACCAG
Genotyping *Zipk*-2-R	AACCCAATGGCTCACTCATCAG
Primers-qRT-PCR	5'-3'
Human *ZIPK*-F	GCACGACATCTTCGAGAACAA
Human *ZIPK*-R	CTTAGAGTGCAGGTAGTGAACG
Human *DEDD*-F	GGAGACATCAATTCGCTATGTGA
Human *DEDD*-R	GCAACACACCACAGGATAGTG
Human *ACTB*-F	AGGATTCCTATGTGGGCGAC
Human *ACTB*-R	ATAGCACAGCCTGGATAGCAA
Mouse *Zipk*-F	ACATTCAGGCAAGAGGATGTTG
Mouse *Zipk*-R	CTCACCTCGCGTTCGATCT
Mouse *Dedd*-F	CACCGCATGTTCGACATCG
Mouse *Dedd*-R	GAAGTCACGTCCATTTCGGAT
Mouse *Actb*-F	GTGACGTTGACATCCGTAAAGA
Mouse *Actb*-R	GCCGGACTCATCGTACTCC

Notes: F, Forward primer. R, Reverse primer.

Tabel S4. Scoring rules and scores of the modified neurological severity score (mNSS).

Task	Description	Points
Tail lift test	Forelimb flexion	1
	Hind limb flexion	1
Walking test	Walk normally	0
	Unable to walk in a straight line	1
Round balancing (7 mm stick)	a. Mice are able to balance on a balance bar	0
	b. Mice cling to the bar	1
	c. One side of the front and back limbs clings to the bar, and the contralateral limb falls off the bar	2
	d. Mice hold the bar and either limbs dropped or rotated while holding the bar for more than 30 seconds	3
	e. Mice try to balance but eventually fail and fall off the bar within 20-30 seconds	4
	f. Mice try to balance but eventually fail and fall off the bar within 10-20 seconds	5
	g. Mice fell directly off the bar within 10 seconds	6
Task	Description	Points (success/ failure)
Beam walk: 3 cm	Able to cross a 30-cm long beam of 3 cm width in 3 min	0/1
Beam walk: 2 cm	Same task but with increased difficulty on a 2 cm wide beam	0/1
Beam walk: 1 cm	Same task but with increased difficulty on a 1 cm wide beam	0/1
Maximum score		12

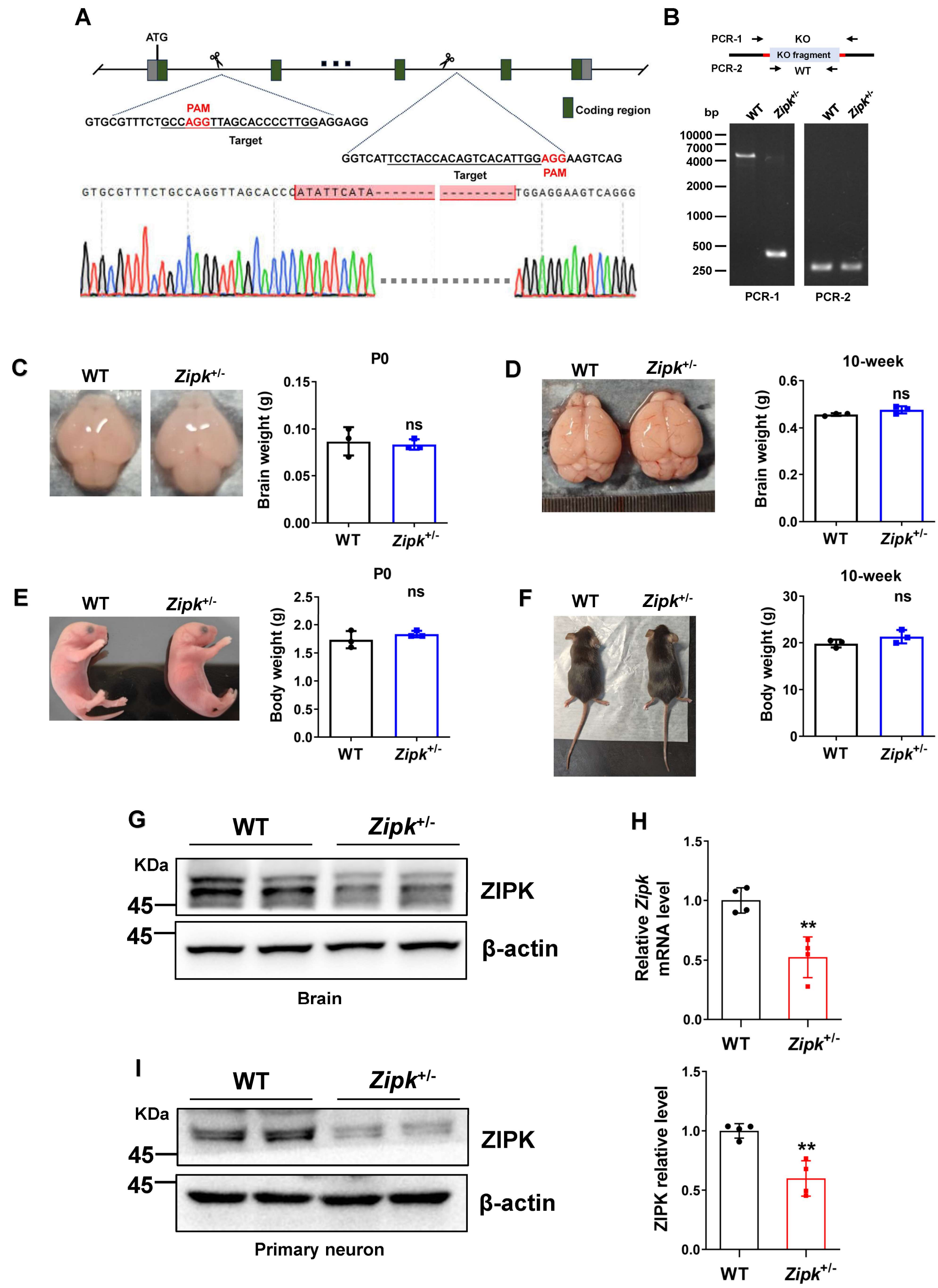

Fig. S1 Generation of *Zipk*$^{+/-}$ mice via the CRISPR/Cas9 system. (A) The guide RNA target sites are underlined, and the PAM sequences are marked in red. Gene sequencing after editing is shown below. (B) Representative genotyping of WT and *Zipk*$^{+/-}$ mice. (C-D) Brain morphology and weight of newborn and 10-week-old WT and *Zipk*$^{+/-}$ mice. (E-F) The

appearance and body weight of newborn and 10-week-old WT and *Zipk*$^{+/-}$ mice. (G-H) Measurement of ZIPK protein and mRNA levels in brain tissues from 3-month-old WT and *Zipk*$^{+/-}$ mice. (I) Immunoblot analysis of ZIPK protein level in WT and *Zipk*$^{+/-}$ primary neurons. $**p < 0.01$, ns, not significant. Unpaired two-tailed Student's *t*-test was used for analysis.

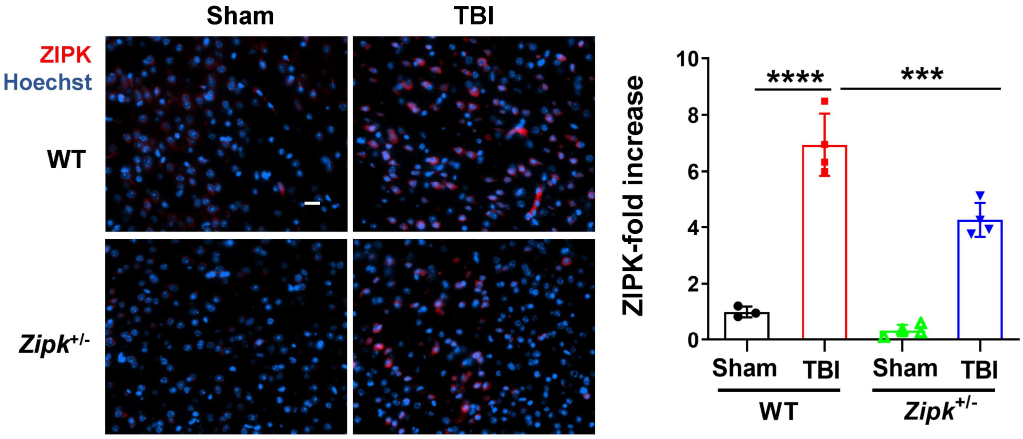

Fig. S2 The expression of ZIPK in peri-injury sites of WT and *Zipk*$^{+/-}$ mice after TBI. WT and *Zipk*$^{+/-}$ mice were subjected to CCI and brain samples were collected at 16 days post TBI. Immunofluorescence analysis was performed to analyze the protein expression of ZIPK in peri-injury brain regions. n=3-4 mice/group. The scale bar is 20 μm. $***p < 0.001$, $****p < 0.0001$. One-way ANOVA was used for comparison.

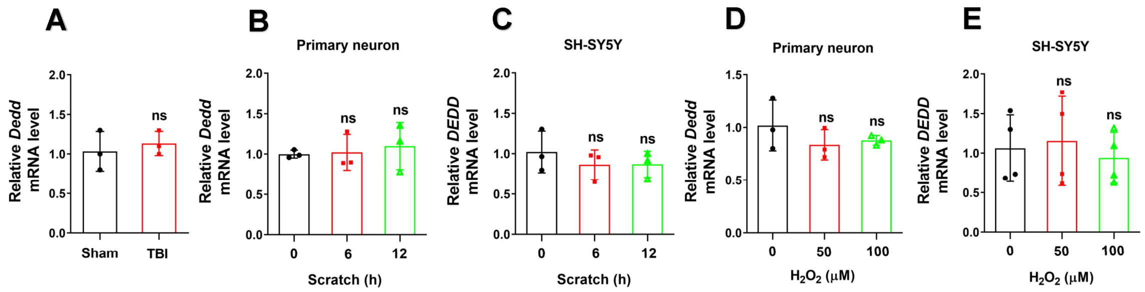

Fig. S3 *DEDD* mRNA levels in *in vivo* and *in vitro* TBI models. qRT-PCR was used to determine *DEDD* mRNA levels in mouse (A) and cellular (B-E) TBI models. Unpaired two-tailed Student's *t*-test was used in A. One-way ANOVA was used in B-E. ns, not significant.

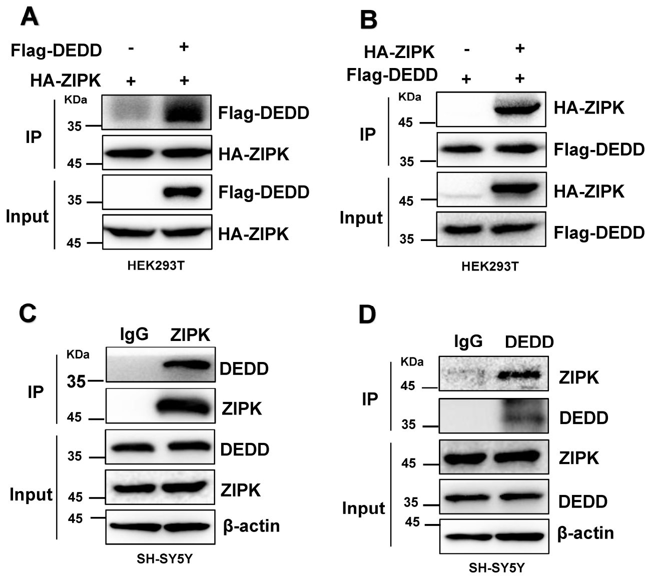

Fig. S4 ZIPK binds to DEDD. (A-B) Representative immunoblots of Flag-DEDD and HA-ZIPK in HEK293T cells transiently transfected with Flag-DEDD and HA-ZIPK constructs in Co-IP assays. Anti-HA or -Flag antibody was used for immunoprecipitation, respectively. (C-D) Endogenous binding of ZIPK and DEDD in SH-SY5Y cells using anti-ZIPK or -DEDD antibody in Co-IP assays.

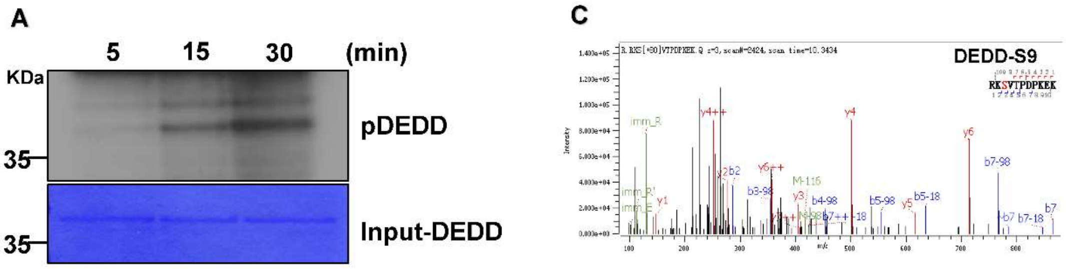

B

Position	Peptide (ProteinMetricsConfidential)	Observed m/z	Mass error (ppm)	Score	Scan time	Intensity
9	R.RAS[79.996]QVWPEEHGEQEHGLYSLHR.M	682.063	0.7	632.6	63.4113	169420000
132	R.ALS[+79.996]DPEPRPPQPSK.T	799.882	-0.9	750.0	38.8067	532260000
182	K.RKS[+79.996]VTPDPKEK.Q	455.570	1.4	475.3	10.3434	28640000

Fig. S5 ZIPK directly phosphorylates DEDD. (A) Representative ^{32}P-autoradiography data showing ZIPK-induced DEDD phosphorylation in a time dependent manner in the *in vitro* kinase assay. (B) Candidate phosphorylation sites in DEDD according to mass spectrometry analysis. (C) Secondary diagram of the DEDD-S9 phosphorylation according to mass spectrometry.

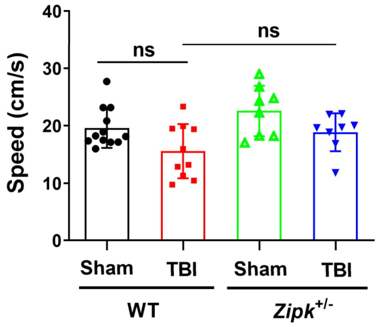

Fig. S6 The swimming speed of four groups of mice in the Morris water maze test. n=8-12 mice/group. ns, not significant. One-way ANOVA was used for analysis.
