# Supplementary figures and images for: Zipper-interacting protein kinase mediates neuronal cell death and cognitive dysfunction in traumatic brain injury via regulating DEDD
Source: Cell Death Dis. 2025 Mar 4;16(1):151. doi: 10.1038/s41419-025-07474-7 (PMC11876612; doi:10.1038/s41419-025-07474-7)

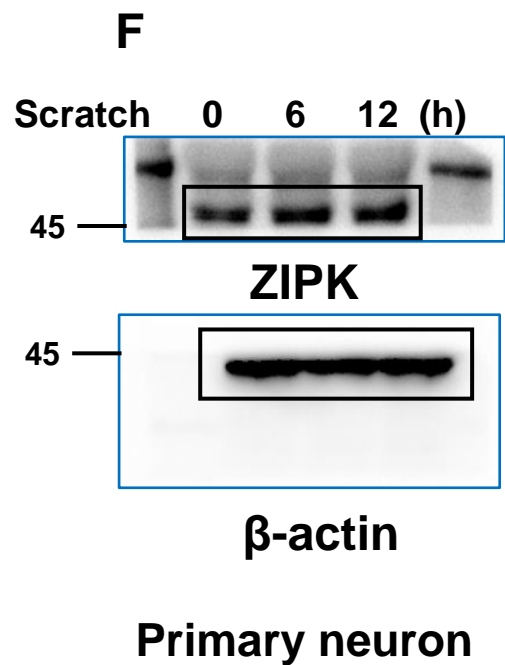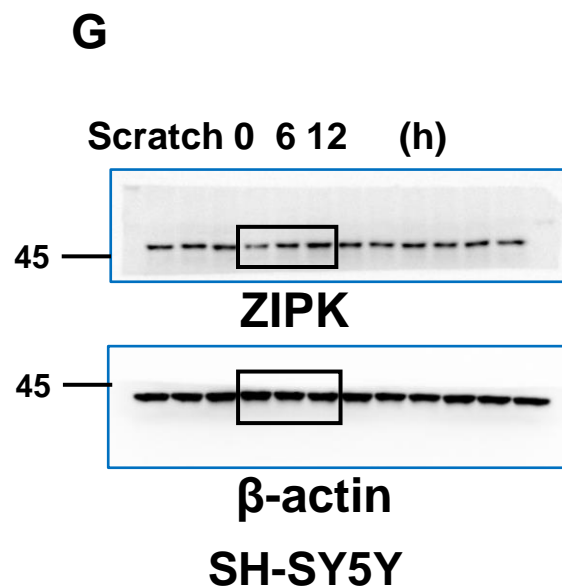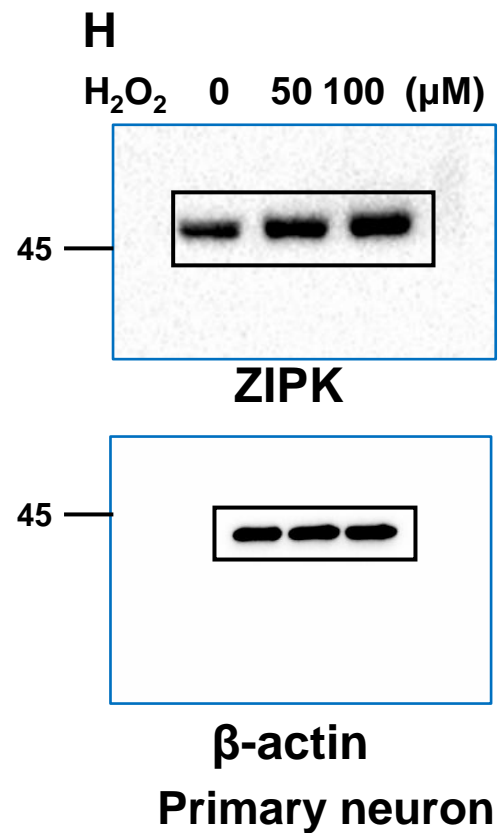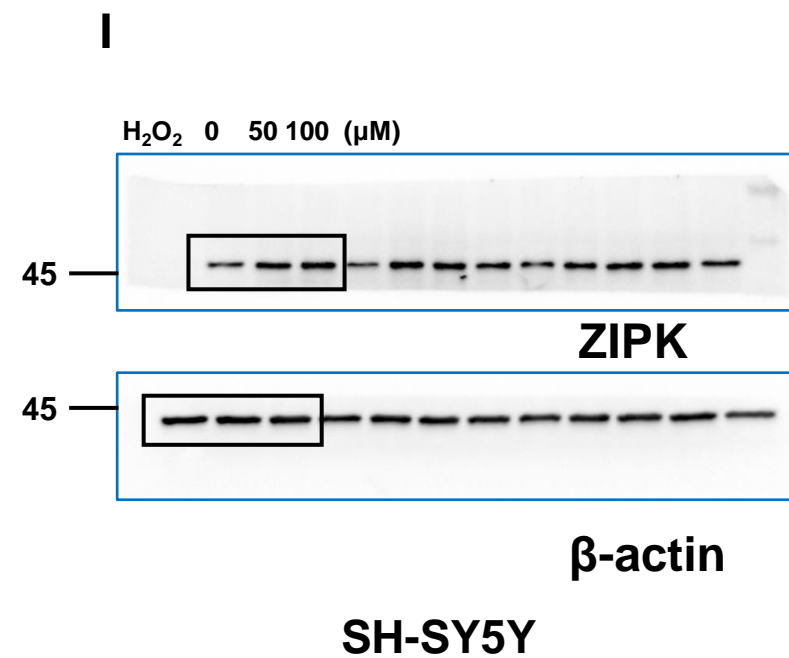

Figure 1

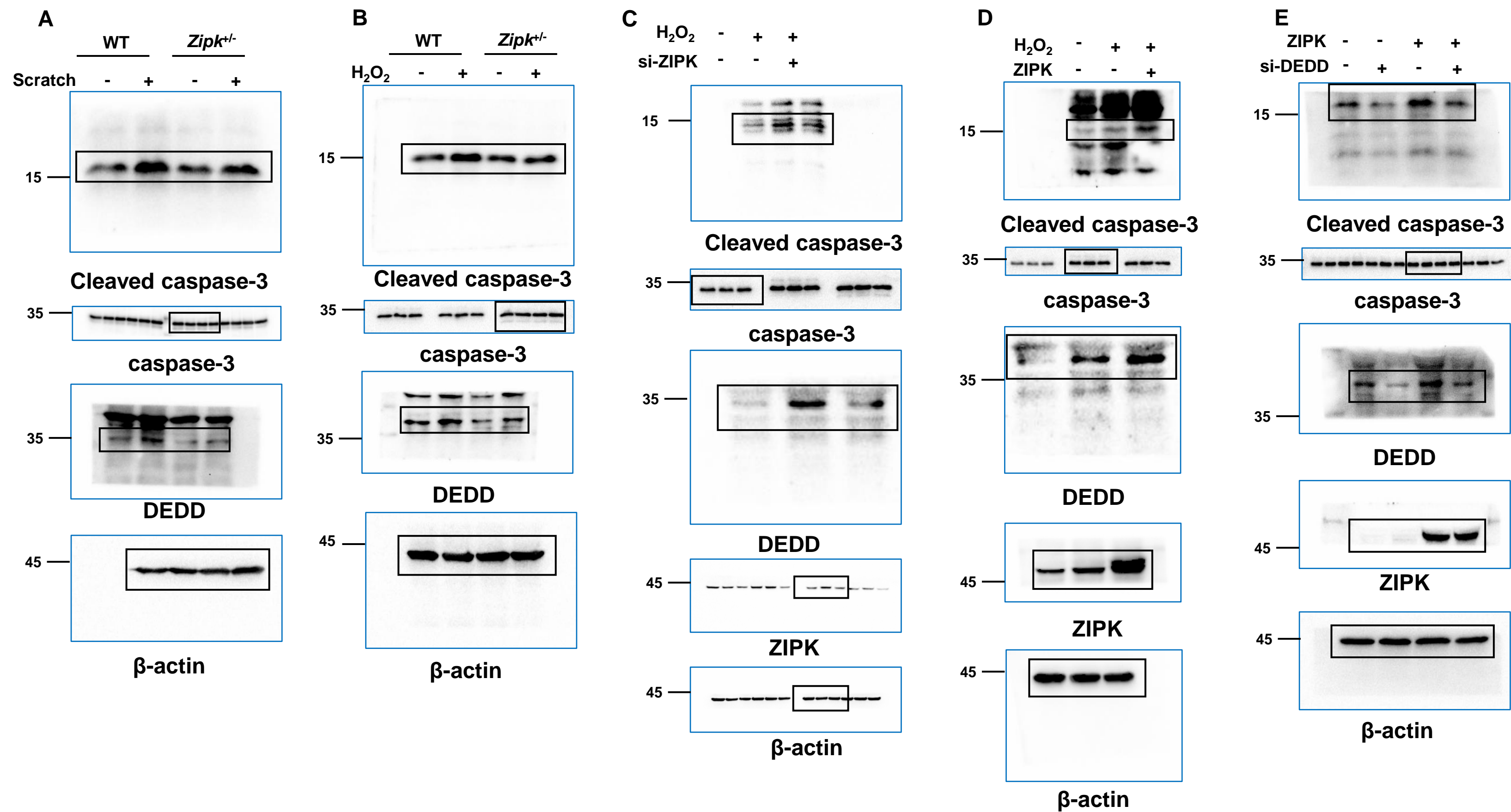

Figure 3

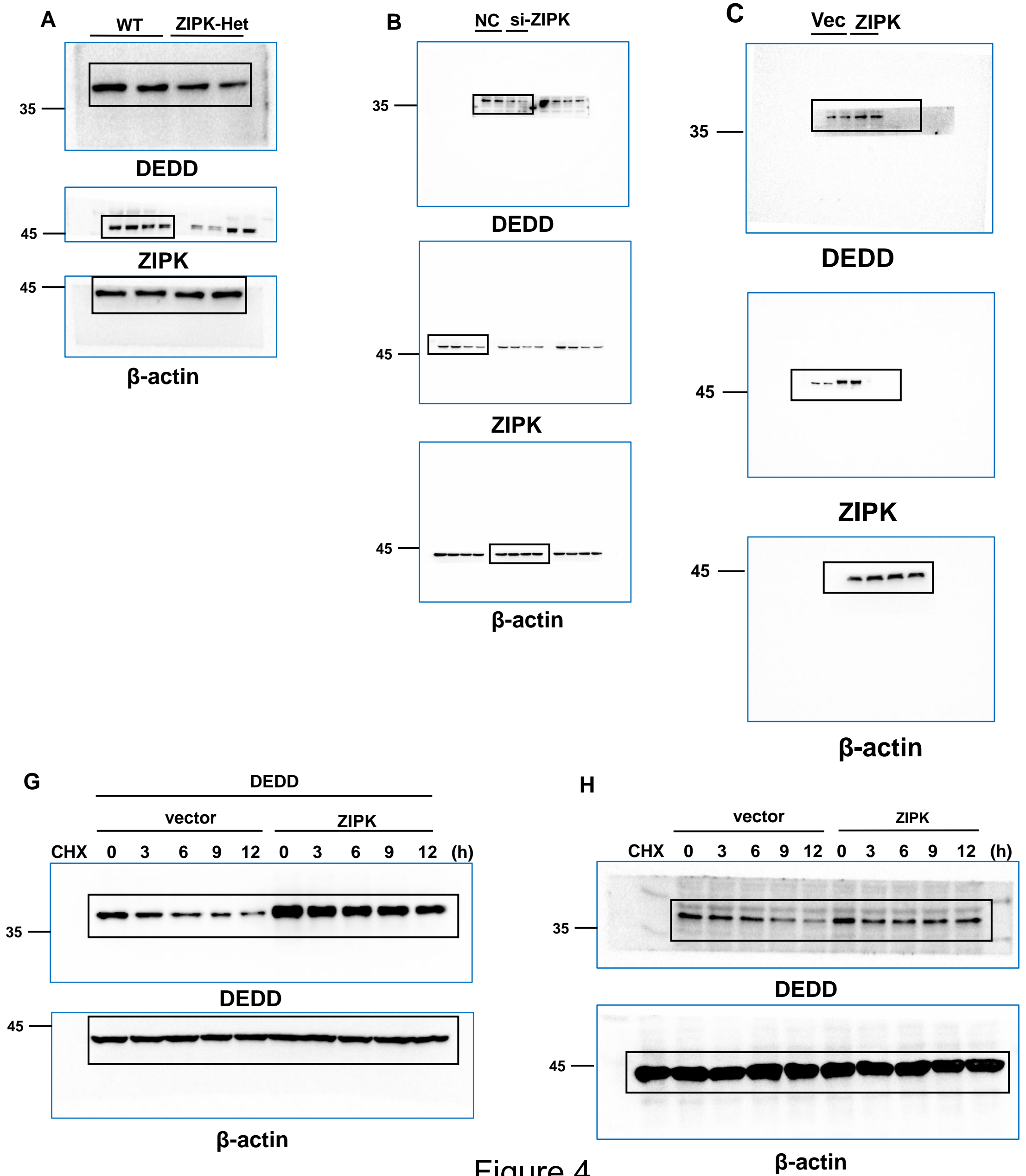

Figure 4

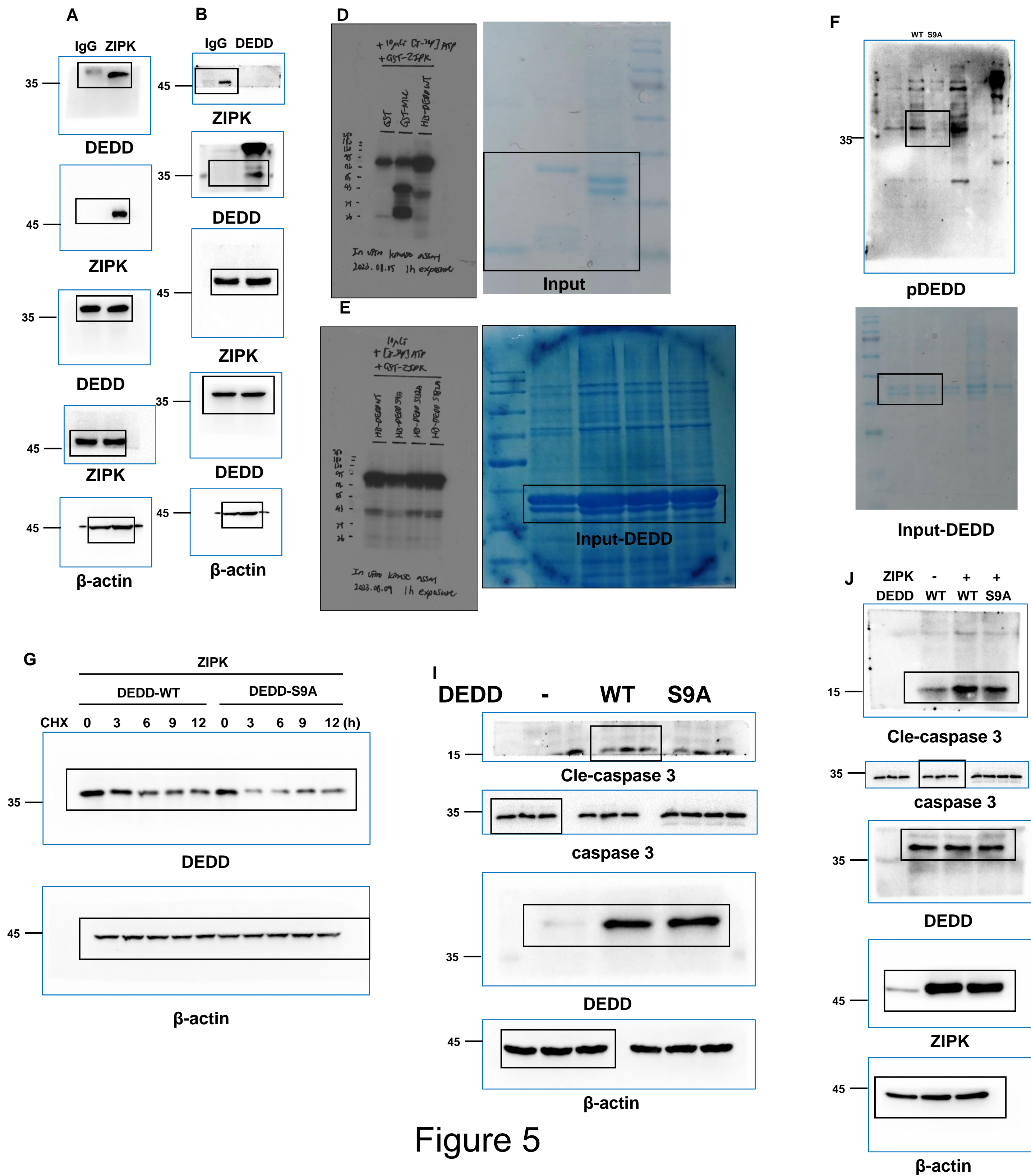

Figure 5

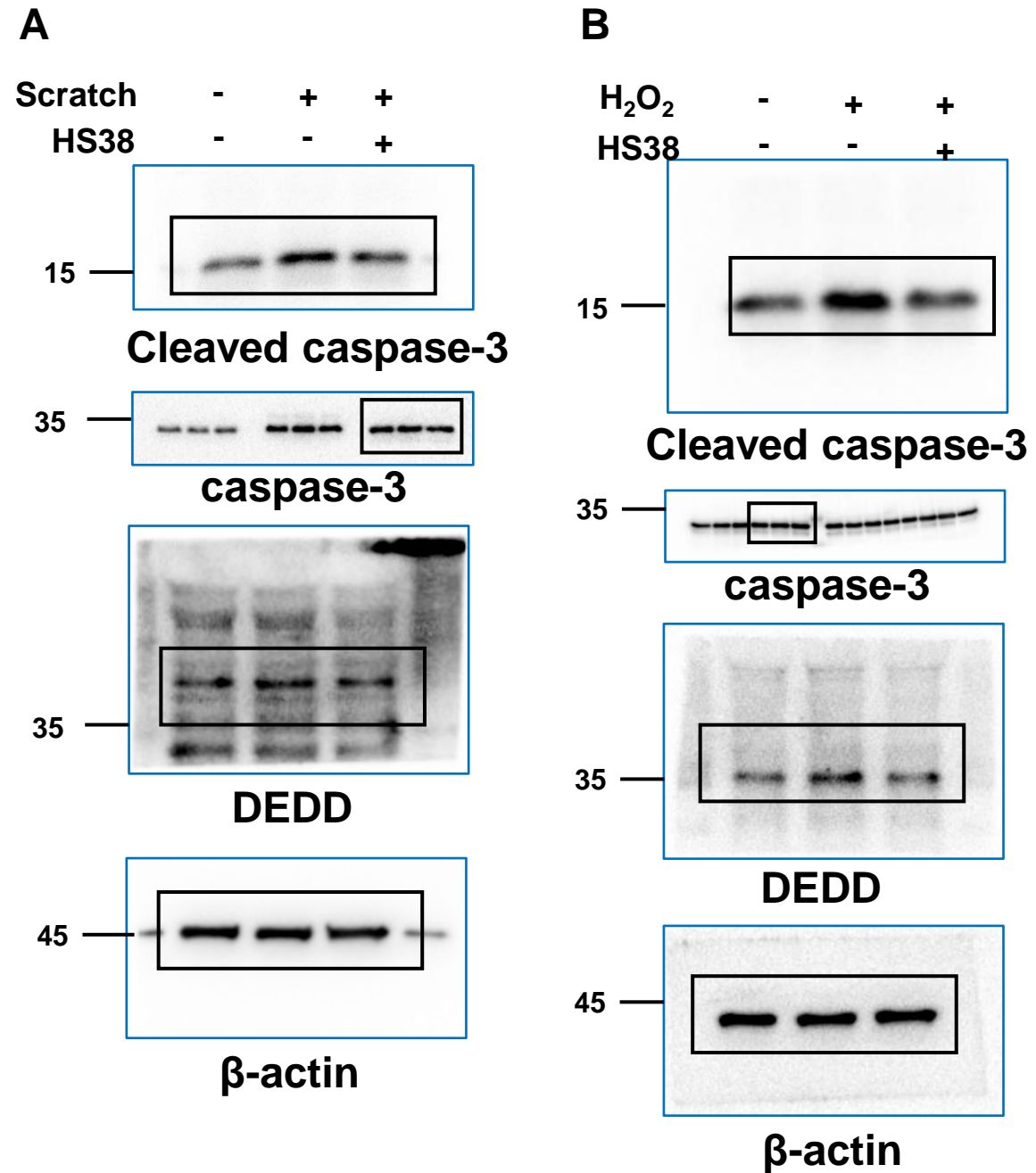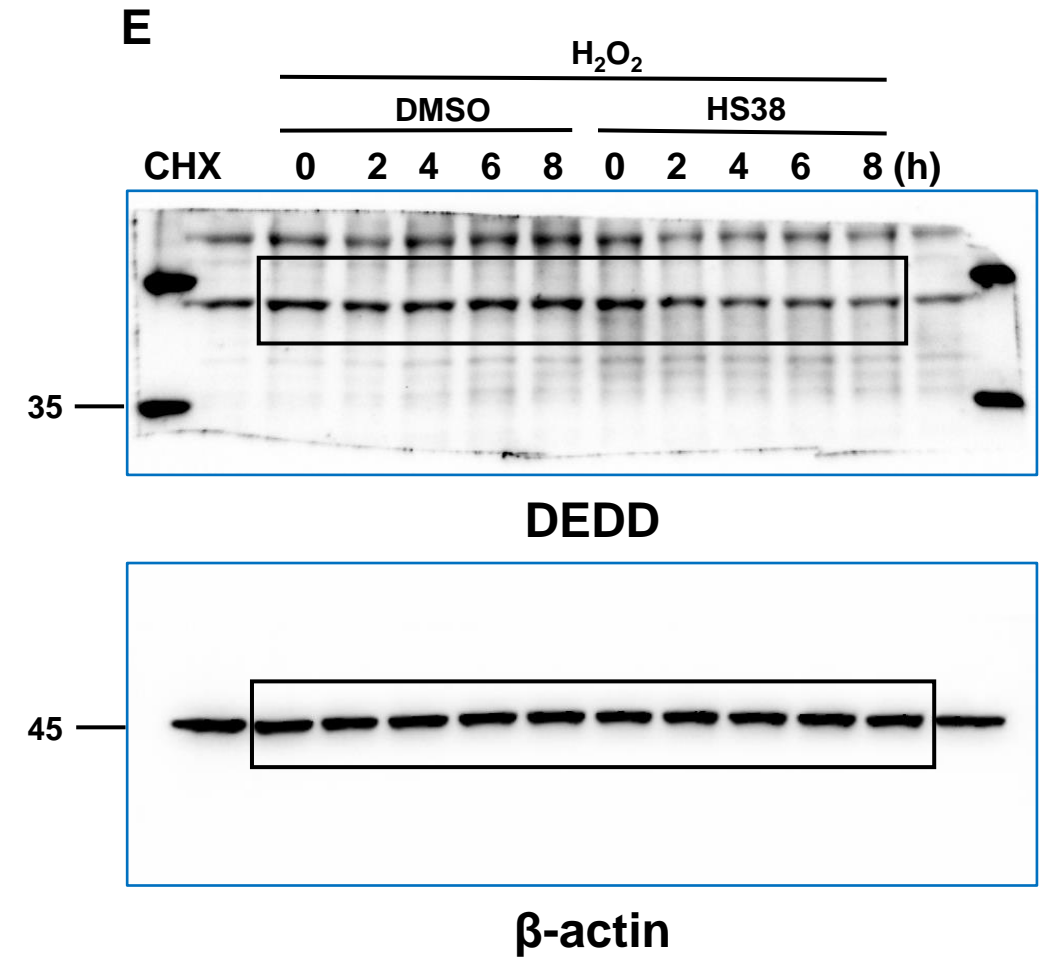

Figure 8

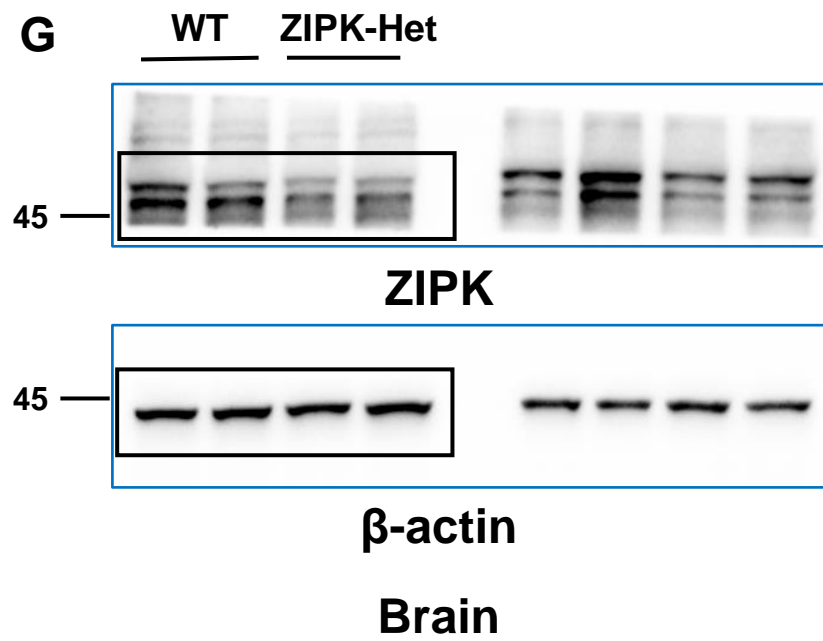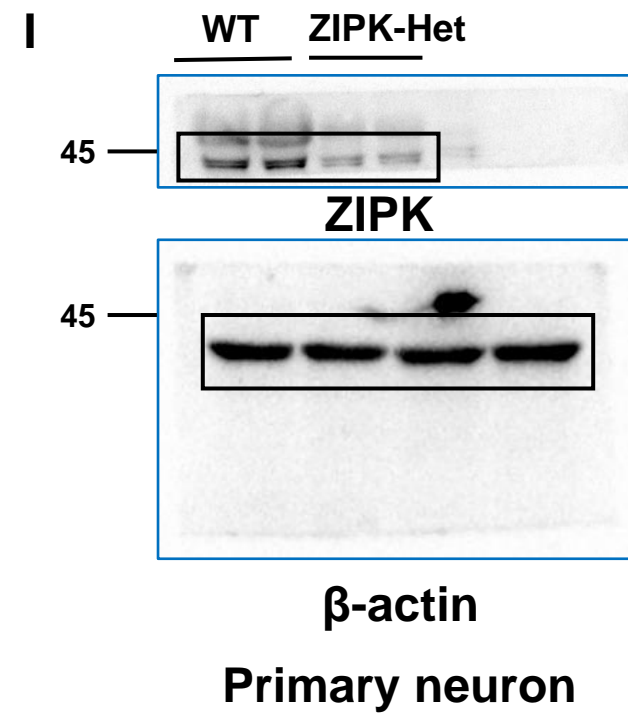

Figure S1

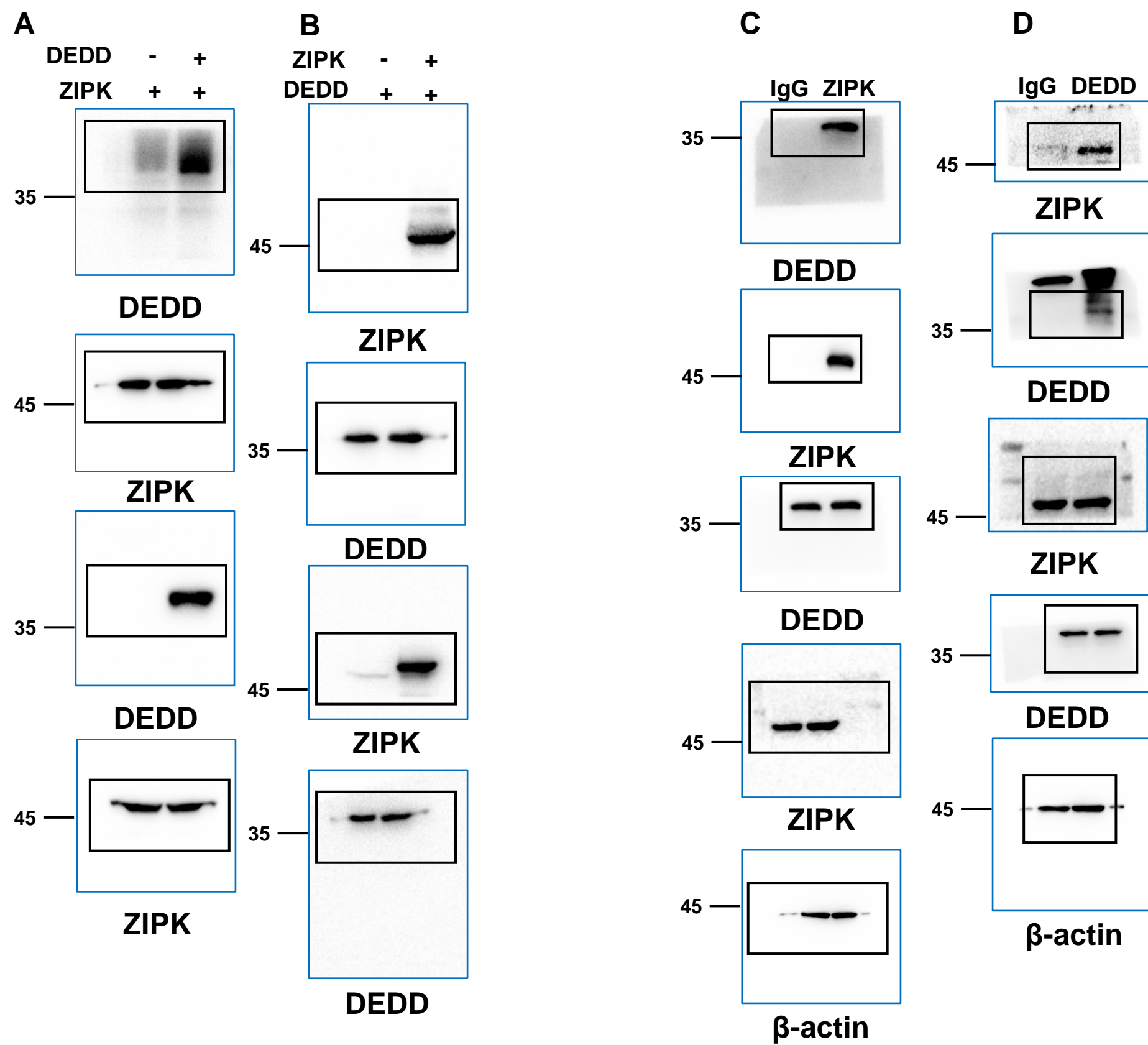

Figure S4

A

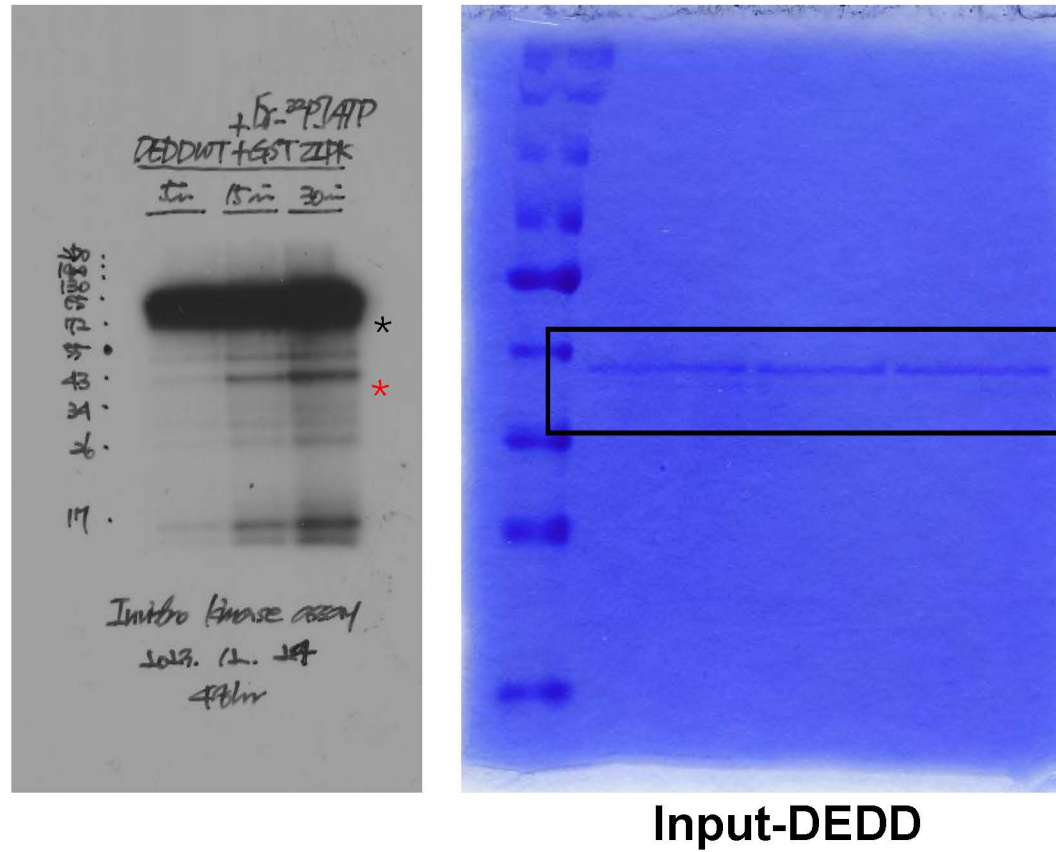

Figure S5

Supplement: Supplementary file 2 — Full and uncropped Western blots [file 41419_2025_7474_MOESM2_ESM.pdf]
